# An ovine septic shock model of live bacterial infusion

**DOI:** 10.1186/s40635-024-00684-x

**Published:** 2024-10-28

**Authors:** Nchafatso G. Obonyo, Sainath Raman, Jacky Y. Suen, Kate M. Peters, Minh-Duy Phan, Margaret R. Passmore, Mahe Bouquet, Emily S. Wilson, Kieran Hyslop, Chiara Palmieri, Nicole White, Kei Sato, Samia M. Farah, Lucia Gandini, Keibun Liu, Gabriele Fior, Silver Heinsar, Shinichi Ijuin, Sun Kyun Ro, Gabriella Abbate, Carmen Ainola, Noriko Sato, Brooke Lundon, Sofia Portatadino, Reema H. Rachakonda, Bailey Schneider, Amanda Harley, Louise E. See Hoe, Mark A. Schembri, Gianluigi Li Bassi, John F. Fraser

**Affiliations:** 1 Critical Care Research Group, The Prince Charles Hospital, 627 Rode Road, Level 3 Clinical Sciences Building, Chermside, Brisbane, QLD 4032 Australia; 2 Institute for Molecular Bioscience (IMB), The University of Queensland, Brisbane, QLD Australia; 3 KEMRI-Wellcome Trust Research Programme and Initiative to Develop African Research Leaders, Kilifi, Kenya; 4 Wellcome Trust Centre for Global Health Research, Imperial College London, London, UK; 5 Children’s Intensive Care Research Program, Child Health Research Centre, Faculty of Medicine, The University of Queensland, Brisbane, QLD Australia; 6 Paediatric Intensive Care Unit, Queensland Children’s Hospital, Brisbane, QLD Australia; 7 School of Chemistry and Molecular Biosciences, The University of Queensland, Brisbane, QLD Australia; 8 Australian Infectious Diseases Research Centre, The University of Queensland, Brisbane, QLD Australia; 9 School of Veterinary Science, Faculty of Science, University of Queensland, Gatton, QLD Australia; 10 Australian Centre for Health Services Innovation and Centre for Healthcare Transformation, Queensland University of Technology, Brisbane, Queensland Australia; 11 Department of Intensive Care, North Estonia Medical Centre, Tallinn, Estonia; 12 Intensive Care Unit, St. Andrew’s War Memorial Hospital, Brisbane, QLD Australia; 13 Faculty of Medicine, The University of Queensland, Brisbane, QLD Australia; 14 Queensland Paediatric Sepsis Program, Children’s Health and Youth Network, Children’s Health Queensland, Brisbane, Queensland Australia; 15 Critical Care Nursing Management Team, Queensland Children’s Hospital, Brisbane, QLD Australia; 16 School of Nursing, Midwifery and Social Work, University of Queensland, Brisbane, QLD Australia

**Keywords:** Haemodynamic monitoring, Resuscitation, *Escherichia coli*, ST131, Animal model, Preclinical

## Abstract

**Background:**

*Escherichia coli* is the most common cause of human bloodstream infections and bacterial sepsis/septic shock. However, translation of preclinical septic shock resuscitative therapies remains limited mainly due to low-fidelity of available models in mimicking clinical illness. To overcome the translational barrier, we sought to replicate sepsis complexity by creating an acutely critically-ill preclinical bacterial septic shock model undergoing active 48-h intensive care management.

**Aim:**

To develop a clinically relevant large-animal (ovine) live-bacterial infusion model for septic shock.

**Methods:**

Septic shock was induced by intravenous infusion of the live antibiotic resistant extra-intestinal pathogenic *E. coli* sequence type 131 strain EC958 in eight anesthetised and mechanically ventilated sheep. A bacterial dose range of 2 × 10^5^–2 × 10^9^ cfu/mL was used for the dose optimisation phase (*n* = 4) and upon dose confirmation the model was developed (*n* = 5). Post-shock the animals underwent an early-vasopressor and volume-restriction resuscitation strategy with active haemodynamic management and monitoring over 48 h. Serial blood samples were collected for testing of pro-inflammatory (IL-6, IL-8, VEGFA) and anti-inflammatory (IL-10) cytokines and hyaluronan assay to assess endothelial integrity. Tissue samples were collected for histopathology and transmission electron microscopy.

**Results:**

The 2 × 10^7^ cfu/mL bacterial dose led to a reproducible distributive shock within a pre-determined 12-h period. Five sheep were used to demonstrate consistency of the model. Bacterial infusion led to development of septic shock in all animals. The baseline mean arterial blood pressure reduced from a median of 91 mmHg (71, 102) to 50 mmHg (48, 57) (*p* = 0.004) and lactate levels increased from a median of 0.5 mM (0.3, 0.8) to 2.1 mM (2.0, 2.3) (*p* = 0.02) post-shock. The baseline median hyaluronan levels increased significantly from 25 ng/mL (18, 86) to 168 ng/mL (86, 569), *p* = 0.05 but not the median vasopressor dependency index which increased within 1 h of resuscitation from zero to 0.39 mmHg^−1^ (0.06, 5.13), *p* = 0.065, and. Over the 48 h, there was a significant decrease in the systemic vascular resistance index (*F* = 7.46, *p* = 0.01) and increase in the pro-inflammatory cytokines [IL-6 (*F* = 8.90, *p* = 0.02), IL-8 (*F* = 5.28, *p* = 0.03), and VEGFA (*F* = 6.47, *p* = 0.02)].

**Conclusions:**

This critically ill large-animal model was consistent in reproducing septic shock and will be applied in investigating advanced resuscitation and therapeutic interventions.

**Supplementary Information:**

The online version contains supplementary material available at 10.1186/s40635-024-00684-x.

## Introduction

Preclinical models are crucial to advance the understanding of disease pathophysiology and to test new treatments. However, there has been limited clinical translation of resuscitation strategies from animal models of sepsis. The lack of a reproducible, and clinically relevant sepsis model has been one of the key translational barriers. Preclinical replication of the complexity of clinically relevant bacterial infection, host immune responses and acute-phase intensive care resuscitation in existing small and large animal models has been challenging. The recent minimum quality threshold in preclinical sepsis studies (MQTiPSS) guidelines recommended the use of live pathogens replicating human disease for preclinical sepsis modelling [1, 2]. The major bacterial pathogens associated with clinical sepsis include *Escherichia coli*, *Klebsiella pneumoniae*, *Staphylococcus aureus*, *Streptococcus pyogenes* and *Streptococcus pneumoniae* [3]*. E. coli* is responsible for ~ 25% of all bloodstream infections and > 50% of all episodes of gram-negative bacterial infections [4] and is often reported as the most common cause of human bacterial sepsis [5, 6]. Translation of resuscitative therapies found to be effective in smaller animals has also been another challenge of preclinical models. While recently, there has been development of a few large animal models of live bacterial sepsis, challenges, such as the lack of sufficient standardization in the severity of injury, duration of sepsis induction, and resuscitation protocols remain [2, 7, 8]. The effects of live bacterial infection on the endothelial–glycocalyx vascular lining and its impact on microvascular dysfunction, and dissociation of tissue oxygen delivery versus consumption remain unclear. One of the most common interventions in septic shock resuscitation, the administration of fluid boluses, lacks conclusive evidence and has been associated with increased harm in recent studies [9–14]. A resuscitation strategy involving early vasopressor use in septic shock has been associated with shock control within 6 h, a lower requirement for fluid boluses and a reduced risk of death in retrospective and small empirical studies [15, 16]. However, the adoption of this practice lacks empirical support from extensive randomized controlled trials in critically ill patients and warrants pre-clinical testing [17]. Given these knowledge gaps, we aimed to develop an ovine model of septic shock, induced by clinically relevant live extra-intestinal pathogenic *E. coli,* resuscitated using strict standardised protocols. Additionally, we aimed to describe the endothelial–glycocalyx and microcirculatory function in the first 48 h of shock resuscitation.

## Methods

### Ethics

The study was approved by the Office of Research Ethics and Integrity of The Queensland University of Technology (QUT approval number 2021000185) and ratified by The University of Queensland (UQ ratification number 2021/AE000607). The study was conducted in accordance with the Australian Code of Practice for the Care and Use of Animals for Scientific Purposes [18] as well as the Critical Care Research Group’s (CCRG) and QUT–Medical Engineering Research Facility (QUT–MERF) Standard Operating Procedures (SOP) [19].

### Bacterial preparation

An inoculum of *E. coli* sequence type 131 (ST131) strain EC958 was prepared from an overnight culture grown in Luria Bertani broth. EC958 is non-susceptible to antibiotics from multiple classes, including third-generation cephalosporins, fluroquinolones, aminoglycosides, tetracyclines [20–22]. Importantly for our infection model, EC958 is susceptible to gentamicin but non-susceptible to cefazolin (a first-generation cephalosporin). The culture was centrifuged, washed, and resuspended in gluconate/acetate-buffered solution (140 mM sodium, 23 mM gluconate, 27 mM acetate, 5 mM potassium, 1.5 mM magnesium, 98 mM chloride, pH 7.4; Baxter). The cell suspension was adjusted to an optical density at 600 nm (OD_600nm_) = 0.014 [corresponding to 2 × 10^7^ colony forming units (cfu)/mL], OD_600nm_ = 0.00014 (2 × 10^5^ cfu/mL), or OD_600nm_ = 1.4 (2 × 10^9^ cfu/mL). A total volume of 300 mL was prepared in six aliquots of 50 mL and used for infection. The bacterial inoculum resuspended in gluconate/acetate-buffered solution was stable with no significant loss of viability at room temperature over 36 h, as evaluated by enumeration of colony forming units at 12-, 24-, and 36-h post-preparation of the inoculum by serial dilution in gluconate/acetate-buffered solution and plating on LB agar.

### Animal preparation

Eight Merino-Dorset first cross ewes (1–2 years) were used in these experiments; four for bacterial dose optimisation and four in the model development. All animals were sourced from Warwick Saleyards and housed at QUT–MERF, with ad libitum access to proprietary sheep feed, lucerne, water, and shade. Prior to the study, the animals underwent overnight fasting and were taken into the operative theatre on a custom-made restraining trolley on the morning of the experiment. A central venous cannula was inserted into the left external jugular vein and the animals were sedated with midazolam 0.5 mg/kg, induced with propofol (3–5 mg/kg) for orotracheal intubation prior to being connected to a mechanical ventilator (Hamilton-G5, Hamilton Medical, Switzerland). At the start of the experiment, the mechanical ventilatory parameters were set as follows; volume-controlled mode, tidal volume of 8 mL/kg, positive end-expiratory pressure (PEEP) of 5 cmH_2_O (0.49 kPa), respiratory rate adjusted to maintain arterial partial pressure of carbon dioxide (PaCO_2_) at 40 ± 5 mmHg (5.3 ± 0.7 kPa), and inspiratory fraction of inspired oxygen (FiO_2_) adjusted to maintain arterial partial pressure of oxygen (PaO_2_) > 70 mmHg (9.3 kPa). Anaesthesia was maintained using midazolam (0.5–0.7 mg/kg/h), fentanyl (20–50 mcg/kg/h), and ketamine (3–8 mg/kg/h). All animals received up to 500 mL Hartmann’s solution (guided by echocardiography) to offset losses during overnight fasting, followed by a continuous maintenance infusion of 1 mL/kg/h and haemodynamic management and continuous monitoring. The animals underwent instrumentation, including urinary catheter and orogastric tube insertion, invasive blood pressure monitoring, pulmonary artery catheter (Swan-Ganz) insertion, and surgical tracheostomy as previously described [19, 23].

### Experimental protocol

**Fig. 1:**
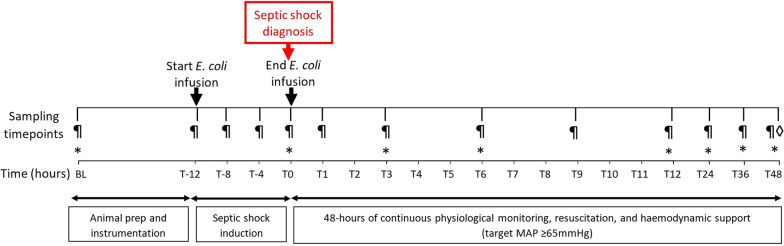
Experimental timeline and sampling schedule; pilcrow sign: blood samples; asterisk: microcirculation incident dark field imaging; lozenge: tissue autopsy. *BL* baseline, *MAP* mean arterial pressure, *SaO*_*2*_ arterial oxygen saturation, *ETCO*_*2*_ end-tidal carbon dioxide)

Septic shock was induced by continuous intravenous infusion of live *E. coli* EC958 bacteria up to a maximum bacterial dose of 12 × 10^7^ cfu/kg. A pre-determined time to develop shock of ≤ 12 h was used (Table 1). Dosing and infusion-rate regimes were optimized based on previously published literature [24]. Septic shock was confirmed using the Sepsis-3 clinical criteria [25] (detailed later). A conservative fluid resuscitation strategy involving doubling the maintenance infusion fluid rate was used and noradrenaline infusion was initiated if the mean arterial blood pressure (MAP) dropped to < 65 mmHg for > 5 min. Details of ventilation management, resuscitation, and de-escalation of support are included in Supplemental Tables 1–3. To maintain asepsis prior to instrumentation, all animals received pre-surgical prophylaxis with 8-hourly cefazolin (1 g) from the start of the experiment (T-baseline). Upon confirmation of septic shock (T0) antibiotic treatment was commenced using 12-hourly gentamicin (2 mg/kg) and animals were thereafter monitored for 48 h (T48) with serial blood sampling (Fig. 1). At the end of the experiment, animals were euthanised using intravenous sodium pentobarbitone (0.5 mL/kg) and autopsy performed for organ (heart, lungs, liver, kidney, and brain) retrieval (Supplemental methods for histology and electron microscopy).

**Table 1:** Dose and infusion rate optimisation and model development

(a) Bacterial dose and infusion-rate optimisation				
Experiment number	Bacterial dose (cfu/mL)^a^	Infusion rate (mL/h)^b^	Time to shock (hours)	Survival duration (hours)
1	2.9 × 10^9^ (loading dose) and 1.4 × 10^9^ (maintenance dose)	1.2	2.5	5
2	2.0 × 10^7^	1.2 (doubling infusion rate hourly)	2	8
3	2.0 × 10^5^	1.2 (increased by 0.6 after 12 h)	27	18
4	2.0 × 10^7^	0.3 (increased by 0.3 every hour)	9	12

(b) 48-h model development				
Experiment number	Bacterial dose (cfu/mL)^c^	Infusion rate (mL/h)^b^	Time to shock (hours)	Survival duration (hours)
5	2.0 × 10^7^	1.2 (doubling infusion rate every h)	6	48
6	2.0 × 10^7^	1.2 (doubling infusion rate every h)	10	48
7	2.0 × 10^7^	1.2 (doubling infusion rate every h)	9	48
8	2.0 × 10^7^	1.2 (doubling infusion rate every h)	9	48
9	2.0 × 10^7^	1.2 (doubling infusion rate every h)	11	48

*cfu* colony forming units

^a^The *E. coli* EC958 infection doses tested are shown in cfu/mL (i.e., 2 × 10^5^, 2 × 10^7^ and 2 × 10^9^)

^b^Graduated increases in the infusion rate impacted the time to shock

^c^The *E. coli* EC958 2 × 10^7^ cfu/mL infection dose was optimal for induction of reproducible septic shock within 12 h and survival to 6 h or more

### Physiological monitoring

Mechanical ventilation was assessed using the following variables; driving pressure, PaO_2_/FiO_2_ (P/F) ratio, static compliance, and oxygen saturation. Hemodynamic monitoring was performed by assessing the heart rate, central venous pressure, systemic and pulmonary arterial blood pressures, and systemic vascular resistance and cardiac indices. Hemodynamic support (vasopressor requirement and resuscitation fluid volume) was recorded over time.

### Microcirculatory monitoring

Microcirculatory blood flow was assessed in the sublingual mucosa (CytoCam™, Braedius) for serial quantification of the total and perfused vessel densities.

### Blood and tissue sampling

Arterial blood gas samples were collected for the analysis of serum lactate, pH, and base excess. Full blood counts and differential white cell counts were performed using a BC-5000 Vet Auto hematology analyzer (Mindray, Shenzhen, China). Pro-inflammatory (IL-6, IL-8 and VEGFA) and anti-inflammatory (IL-10) cytokines were analyzed using custom Luminex Magpix-based assays (R&D Systems, MN, USA) in accordance with the manufacturer’s instructions. Tissue samples obtained at autopsy underwent processing and staining for histopathology and transmission electron microscopy.

### Endothelial–glycocalyx assessment

Endothelial–glycocalyx integrity was assessed using enzyme-linked immunosorbent assays (ELISAs) for the biomarker hyaluronan (R&D Systems, Minneapolis, MN, USA) in arterial blood samples collected at specific time points (Fig. 1).

### Outcomes

The primary outcome of this study was the development of septic shock within 12 h of the initiation of *E. coli* infusion. The secondary outcomes included the vasopressor dependency index (VDI), endothelial–glycocalyx integrity (serum hyaluronan), microcirculatory perfusion status (proportion of perfused vessels, total and perfused vessel densities), pro-inflammatory (IL-6, VEGFA), and anti-inflammatory (IL-10) cytokines, coagulopathy, survival to 48-h post-shock and tissue injury.

### Septic shock and vasopressor dependency definitions

Septic shock was defined as a vasopressor (noradrenaline) requirement to maintain MAP ≥ 65 mmHg and lactate ≥ 2 mmol/L in the absence of hypovolemia [25]. Hypovolemia was assessed using a combination of parameters commonly used clinically [26] including the central venous pressure (CVP), pulmonary artery occlusion/wedge pressure, echocardiography, systemic vascular resistance index and urine output (< 0.5 mL/kg/h). Echocardiography was used to guide resuscitation to avoid volume overload and microcirculation perfusion was also monitored during resuscitation and treatment [27]. To quantify the vasoactive requirement objectively, the VDI was calculated as the ratio of the vasoactive inotrope score (VIS) to the MAP [28]. The VIS score incorporates all medications used to support blood pressure and is expressed as [(dopamine dose × 1) + (dobutamine dose × 1) + (adrenaline dose × 100) + (noradrenaline dose × 100) + (vasopressin × 10,000) + (milrinone × 10)], where all doses are expressed as micrograms/kg/min, except vasopressin expressed as U/kg/min [29]. A VDI of zero indicates that no vasopressors were required to maintain a MAP ≥ 65 mmHg.

### Statistical analysis

All analyses were performed using STATA v18 software (STATA Corp®). Data are presented as mean (standard deviation, SD) for normally distributed variables and median (interquartile range, IQR) for non-normally distributed variables. A mixed-effects model with the time of shock confirmation as the reference point was used to compare the effects of bacterial infusion on hemodynamic variables, from baseline to development of shock and on repeated measures after the commencement of hemodynamic support. Statistical significance was set at an alpha value of < 0.05.

## Results

### Dose optimisation and model development

Four sheep with a median weight of 50 kg (48, 54) were used in the dose optimisation phase. Three *E. coli* EC958 infection doses (2 × 10^5^, 2 × 10^7^ and 2 × 10^9^ cfu/mL) were tested and infusion rates optimised (Table 1a). The infection dose correlated directly with the time to the onset of shock. Once the initial dose of 2 × 10^7^ cfu/mL and infusion rate were established, the model was repeated five times to check for consistency of the dose and infusion rate. The median weight of the five sheep used in the final was 51 kg (47, 55). All these animals received 2 × 10^7^ cfu/mL bacterial dose, as a continuous infusion starting at 1.2 mL/h and the infusion rate was doubled every hour to a maximum rate of 153.6 mL/h (total volume infused 150–300 mL and infection dose 6–12 × 10^7^ cfu/kg) (Table 1b).

### Primary outcome

**Fig. 2:**
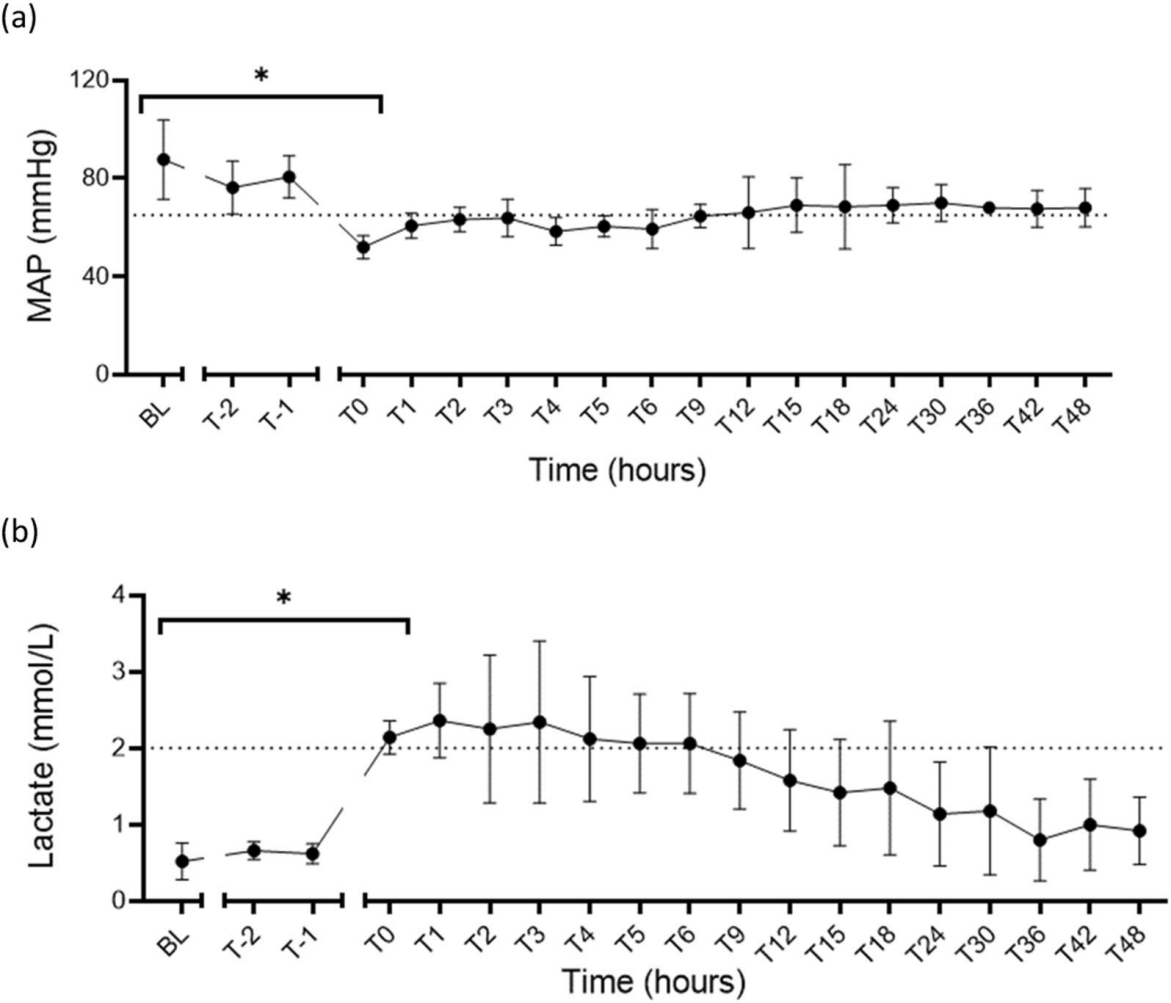
Sepsis-3 criteria showing significant differences from baseline to the time of shock diagnosis in the median (IQR) MAP, reducing from 91 mmHg (71, 102) to 50 mmHg (48, 57), *p* = 0.004 (**a**); and lactate increasing from 0.5 mM (0.3, 0.8) to 2.1 mM (2.0, 2.3), *p* = 0.02 (**b**). *p < 0.05; *MAP* mean arterial pressure, *BL* baseline, *T-2* end of instrumentation, *T-1* start of bacterial infusion, *T0* diagnosis of shock; Dotted lines represent diagnostic cut-off levels of 65 mmHg (MAP) and 2 mM (lactate)

All sheep infected with our optimised 2 × 10^7^ cfu/mL and 1.2 mL/h infusion rate (doubled every hour) developed septic shock and met the Sepsis-3 criteria with a decrease in MAP from a baseline median of 91 mmHg (IQR 71, 102) to 50 mmHg (IQR 48, 57), *p* = 0.004. The baseline lactate levels increased from a median of 0.5 mM (0.3, 0.8) to 2.1 mM (2.0, 2.3), *p* = 0.02 (Fig. 2).

### Secondary outcomes

Bacterial infusion also led to physiological, haemodynamic and laboratory changes consistent with septic shock. Results are described in detail below from the five sheep infected using our optimised protocol.

**Fig. 3:**
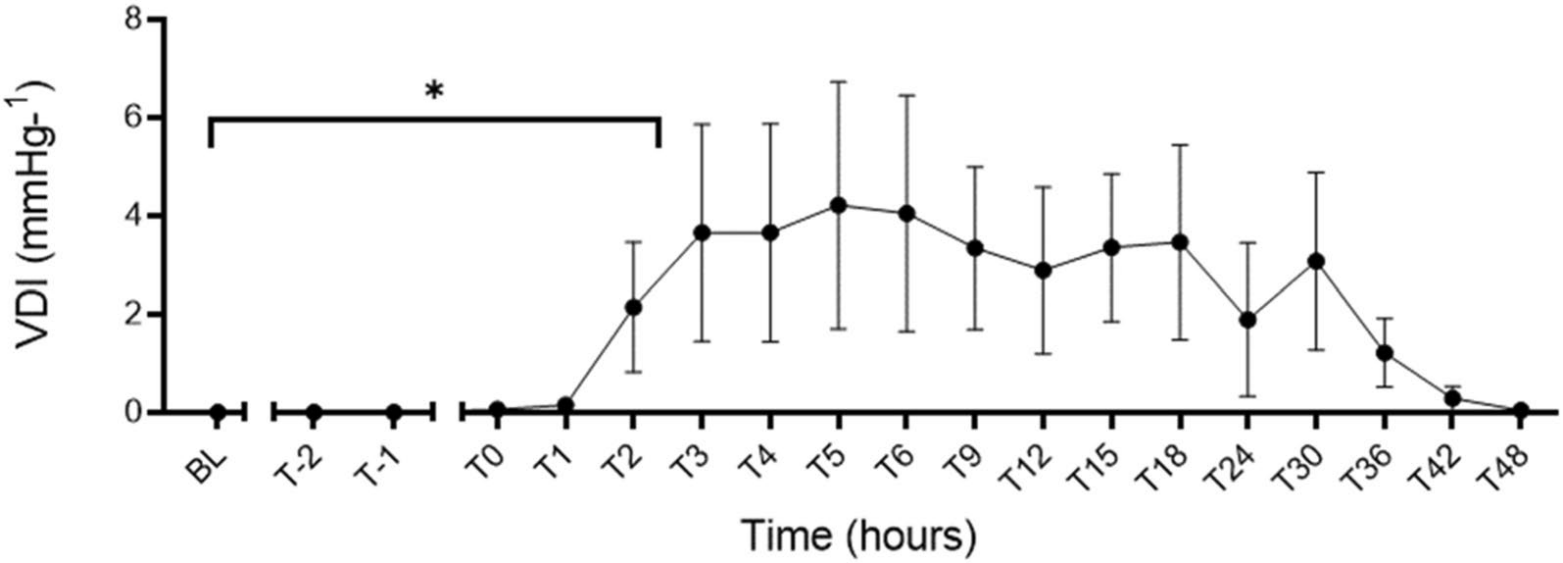
VDI increased from no vasopressor use at baseline to a median of 0.14 mmHg^−1^ (0, 0.35) at the time of shock diagnosis (T0-hours), with a significant increase at T2-hours 1.70 mmHg^−1^ (0.47, 3.96) and a peak at T5-hours 4.52 mmHg^−1^ (1.92, 6.84). *p < 0.05; *VDI* vasopressor dependency index, *BL* baseline, *T-2* end of instrumentation, *T-1* start of bacterial infusion, *T0* diagnosis of shock

- Vasopressor support
  All animals survived to 48-h post-shock confirmation and required vasopressor support to maintain a target MAP (≥ 65 mmHg) with the median VDI rising from zero at baseline to 0.14 mmHg^−1^ (0, 0.35; *p* = 0.34), at time of shock diagnosis (T0-hours), but was non-significant within 1 h of resuscitation, 0.39 mmHg^−1^ (0.06, 5.13), *p* = 0.065. The peak VDI was at T5-hours 5.30 mmHg^−1^ (0.12, 10.66), but had reduced to 0.02 mmHg^−1^ (0, 0.06), *p* = 0.17 by T48-hours (Fig. 3). Similarly, the VIS score increased from a baseline value of zero to a median of 2 units (0, 10) at T0-hours and peaked at T5-hours 219 units (13, 417) but had reduced by T48-hours to 3 units (0, 5), *p* = 0.96 (Table 2). Induction of septic shock also led to acidosis with a significant reduction in base deficit (*p* = 0.02) but not pH (*p* = 0.07) (Table 3).

**Table 2:** Diagnostic criteria and physiological monitoring

	BL	T0	T48	*p* value (BL vs T0)	*p* value (T0 to Tend)
Diagnostic criteria					
MAP (mmHg)	91 (71, 102)	50 (48, 57)	69 (62, 74)	0.004	0.0066
Lactate (mmol/L)	0.5 (0.3, 0.8)	2.1 (2.0, 2.3)	0.7 (0.6, 1.4)	0.02	0.18
Temperature (°C)	37.9 (37.0, 38.3)	38.1 (36.8, 39.9)	39.4 (36.7, 39.8)	0.54	0.10
Vasopressor support					
VDI	0	0.14 (0, 0.35)	0.05 (0, 0.08)	0.34	0.17
VIS	0	2 (0, 10)	3 (0, 5.5)	0.64	0.96
Oxygenation					
*P*/*F* ratio	492(413, 538)	186 (117, 189)	125 (67, 238)	0.03	0.11
Cstat (mL/cmH_2_O)	38 (31, 45)	29 (25, 31)	23 (21, 31)	0.036	0.07
Driving pressure (cmH_2_O)	8.9 (8.4, 12.7)	12.7 (12.0, 14.9)	15.6 (14.0, 17.4)	0.03	0.35
SaO_2_ (%)	100 (99, 100)	93 (89, 99)	96 (86, 97)	0.62	0.98
SvO_2_ (%)	69 (63, 71)	74 (71, 85)	76 (62, 81)	0.88	0.99
Circulation					
HR (beats/min)	87 (76, 93)	108 (96, 114)	75 (69, 95)	0.024	0.04
MPAP (mmHg)	9 (7, 10)	14 (14, 21)	18 (14, 21)	0.02	0.98
PA wedge (mmHg)	2 (2, 6)	8 (7, 11)	12 (8, 14)	0.0041	0.06
CVP (mmHg)	1 (0, 5)	5 (3, 6)	6 (2, 11)	0.35	0.65
CI (L/min/m^2^)	3.5 (3.2, 3.6)	3.8 (2.1, 5.0)	3.5 (3.2, 5.0)	0.99	0.99
SVRI (dynes/cm^5^/m^2^)	1348 (1268, 1448)	676 (483, 1025)	738 (626, 788)	< 0.0001	0.03
Urine output (mL/kg)	0.9 (0.3, 1.8)	0.4 (0.3, 1.8)	0.5 (0.4, 1.1)	0.99	0.32
Fluid balance (mL/kg)	6.3 (2.2, 7.4)	1.8 (1.5, 3.4)	1.3 (0.9, 2.2)	0.13	0.27
Microcirculation					
PPV (%)	38 (28, 92)	31 (18, 53)	42 (34, 62)	0.068	0.82
PVD (mm/mm^2^)	5.6 (3.2, 15.44)	4.2 (2.3, 9.2)	9.5 (6.6, 14.7)	0.17	0.51
TVD (mm/mm^2^)	15.61 (10.2, 18.64)	16.0 (8.53, 17.85)	12.67 (9.72, 13.86)	0.985	0.803

Data presented as medians (IQR); BL, baseline; T0, diagnosis of shock; T48, time of experiment termination in hours; *p* value (BL vs T0), significance test for effect of sepsis induction; *p* value (T0 vs T48), significance test for effect of resuscitation

*MAP* mean arterial pressure, *P/F ratio* ratio of arterial partial pressure of oxygen (PaO_2_) and fraction of inspired oxygen (FiO_2_), *SaO*_*2*_ arterial oxygen saturation, *ScvO*_*2*_ central venous oxygen saturation, *HR* heart rate, *MPAP* mean pulmonary artery pressure, *PA wedge* pulmonary artery wedge pressure, *CVP* central venous pressure, *CI* cardiac index, *SVRI* systemic vascular resistance index, *PPV* proportion of perfused vessels, *PVD* perfused vessel density, *TVD* total vessel density

**Table 3:** Laboratory assessment

	BL	T0	T48	*p* value (BL vs T0)	*p* value (T0 to Tend)
Acidosis					
pH	7.42 (7.39, 7.44)	7.33 (7.32, 7.44)	7.35 (7.31, 7.42)	0.07	0.09
Base deficit (mmol/L)	4.0 (3.5, 5.5)	− 0.9 (− 4.7, 3.0)	− 1.3 (− 2.2, 3.9)	0.02	0.14
Glycocalyx					
Hyaluronan (ng/mL)	25(18, 86)	168 (86, 569)	51 (31, 277)	0.05	0.45
Cytokines					
IL-6 (pg/mL)	8 (8, 49)	12,494 (3143, 18,867)	1650 (1348, 6582)	< 0.01	0.021
IL-8 (pg/mL)	236 (160, 432)	425 (368, 739)	148 (103, 277)	< 0.01	0.03
IL-10 (pg/mL)	36 (22, 146)	1433 (513, 2212)	15 (2, 52)	< 0.01	0.06
VEGFA (pg/mL)	9 (1, 34)	101 (30, 116)	67 (38, 79)	< 0.01	0.08
Coagulopathy					
PT (s)	12 (11, 12)	14 (13, 16)	18 (18, 20)	0.33	0.08
aPTT (s)	30 (24, 35)	46 (30, 48)	53 (37, 68)	0.02	0.04
AT (U/mL)	0.9 (0.8, 0.9)	0.6 (0.5, 0.6)	0.5 (0.4, 0.6)	< 0.01	0.46
Fibrinogen (g/L)	1.4 (1.3, 1.7)	0.9 (0.7, 1.6)	1.4 (1.1, 3.2)	0.05	0.25
Protein C (U/mL)	0.53 (0.41, 0.67)	0.32 (0.26, 0.34)	0.18 (0.13, 0.26)	< 0.01	0.02
Protein S (free) (U/mL)	1.77 (1.50, 1.81)	1.36 (1.19, 1.43)	1.63 (1.50, 1.84)	< 0.01	0.12
ROTEM™					
EXTEM CT (s)	65 (61, 78)	82 (73, 90)	140 (120, 208)	0.07	0.12
INTEM CT (s)	144 (123, 190)	296 (172, 522)	261 (208, 344)	0.36	0.20
FIBTEM CT (s)	61 (55, 68)	74 (67, 82)	124 (106, 180)	0.08	0.18
Multiplate					
ADP 6.5 µM (U)	122 (71, 185)	67 (45, 112)	89 (61, 104)	0.42	0.92
Collagen 3.2 µg/mL (U)	72 (42, 110)	47 (20, 59)	83 (62, 107)	0.41	0.13
End-organ function					
cTnI (ng/mL)	0.06 (0.04, 0.18)	0.54 (0.39, 1.04)	0.57 (0.23, 0.71)	0.03	0.13
ANP (pmol/L)	16 (13, 19)	45 (25, 74)	25 (22, 56)	0.02	0.61
CK (U/L)	162 (157, 170)	2381 (1440, 4561)	779 (226, 1012)	< 0.01	0.04
Creatinine (µmol/L)	90 (80, 109)	93 (73, 109)	113 (90, 234)	0.99	0.18
Total protein (g/L)	67 (64, 73)	46 (41, 48)	37 (34, 40)	< 0.01	< 0.001
Albumin (g/L)	38 (37, 44)	26 (24, 29)	21 (20, 21)	< 0.01	< 0.001
Bilirubin (µmol/L)	2 (2, 4)	7 (3, 8)	11 (5, 13)	0.30	0.16
ALP (U/L)	126 (90, 155)	87 (65, 124)	55 (42, 93)	0.22	0.13
ALT (U/L)	13 (11, 14)	12 (10, 15)	13 (10, 18)	0.99	0.89
AST (U/L)	81 (67, 86)	111 (100, 125)	225 (113, 249)	0.02	0.06
GGT (U/L)	48 (42, 68)	30 (27, 46)	29 (26, 31)	0.03	0.18

Data presented as medians (IQR); BL, baseline; T0, diagnosis of shock; T48, time of experiment termination in hours; *p* value (BL vs T0), significance test for effect of sepsis induction; *p* value (T0 vs T48), significance test for effect of resuscitation

**Fig. 4:**
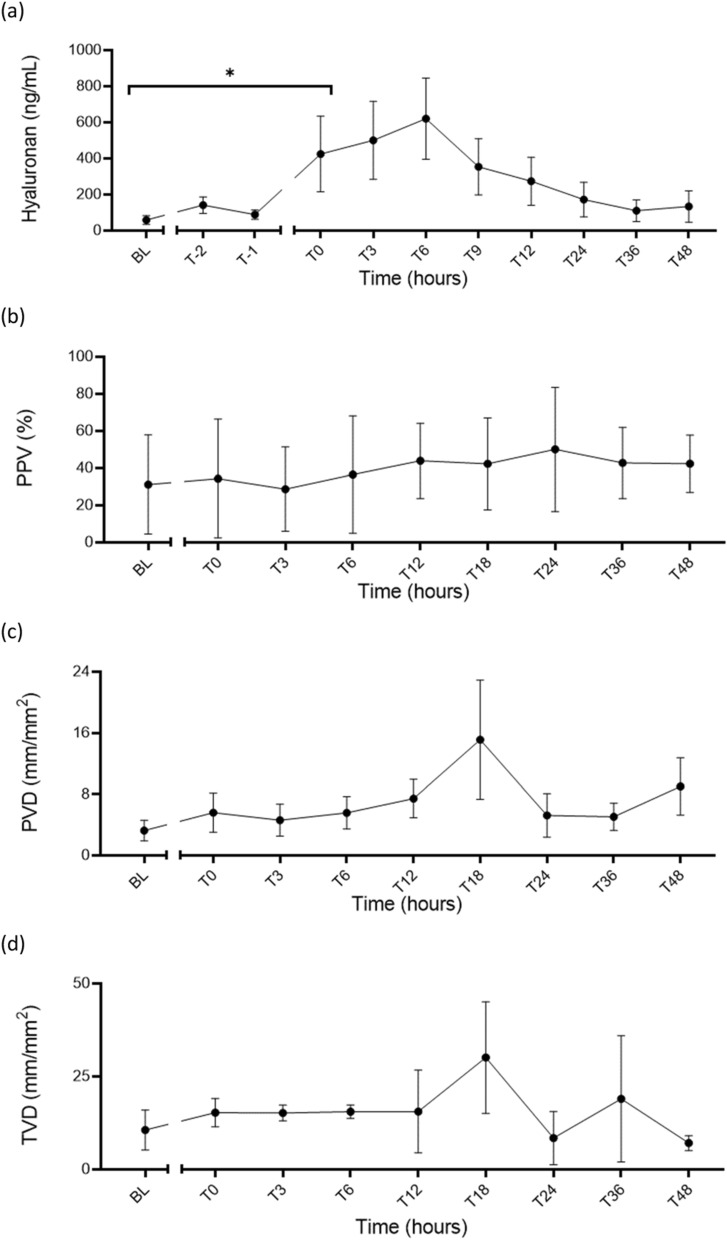
The endothelial biomarker hyaluronan increased significantly from a baseline median of 25 ng/mL (18, 86) to 168 ng/mL (86, 569) after development of septic shock (*p* = 0.05) (**a**). The microcirculation perfusion indices reduced; however, these changes were not statistically significant: PPV (*p* = 0.068) (**b**); PVD (*p* = 0.17) (**c**); and TVD (*p* = 0.985) (**d**). *p < 0.05; *PPV* proportion of perfused vessels, *PVD* perfused vessel density, *TVD* total vessel density, *BL* baseline, *T0* diagnosis of shock
- Physiological monitoring
  (i) Respiratory parameters
    Sepsis induction led to a significant reduction in the *P*/*F* ratio from a median of 492 (413, 538) at baseline to 186 (117, 189) at T0-hours (*p* = 0.03). Similarly, the static lung compliance decreased from a baseline median of 38 mL/cmH_2_O (31, 45) to 29 mL/cmH_2_O (25, 31) at T0-hours (*p* = 0.03). The median pulmonary driving pressure increased from baseline, 8.9 cmH_2_O (8.4, 12.7) (i.e., 0.87 [0.82, 1.25] kPa) to 12.7 cmH_2_O (12.0, 14.9) (i.e., 1.25 [1.18, 1.46] kPa) at T0-hours (*p* = 0.03). Both the median arterial (SaO_2_) and venous (SvO_2_) oxygen saturations decreased from baseline after induction of septic shock, but these changes were non-significant (SaO_2_, *p* = 0.62 and SvO_2_, *p* = 0.88) (Table 2). Over the 48 h of resuscitation and haemodynamic support, there was a significant decline in lung compliance (*F* = 13.69, *p* = 0.004), *P*/*F* ratio (*F* = 5.97, *p* = 0.05) and increase in pulmonary driving pressure (*F* = 8.84, *p* = 0.01) (Fig. 5).

*IL* interleukin, *VEGFA* vascular endothelial growth factor-α, *PT* prothrombin time, *aPTT* activated partial thromboplastin time, *AT* antithrombin, *ROTEM*™ rotational thromboelastometry, *EXTEM* thromboplastin-initiated coagulation, *INTEM* contact-factor-initiated coagulation, *FIBTEM* thromboplastin-initiated coagulation with the platelet inhibitor cytochalasin D, *CT* clotting time, *ADP* adenine diphosphate test, *cTnI* cardiac troponin I, *ANP* atrial-type natriuretic peptide, *CK* creatine kinase, *ALP* alkaline phosphatase, *ALT* alanine aminotransferase, *AST* aspartate aminotransferase, *GGT* gamma-glutamyl transferase
- The microcirculation and endothelial–glycocalyx integrity
  Following sepsis induction, the median proportion of perfused vessels (PPV) reduced from a baseline value of 38% (28, 92) to 31% (18, 53) at T0-hours (*p* = 0.06). Similarly, there was a reduction in the median perfused vessel density (PVD) from 5.6 mm/mm^2^ (3.2, 15.44) at baseline to 4.2 mm/mm^2^ (2.3, 9.2) at T0-hours (*p* = 0.17). Sepsis induction did not lead to a change in the total vessel density (TVD) (Table 2). The serum hyaluronan levels increased significantly from a baseline median value of 25 ng/mL (18, 86) to 168 ng/mL (86, 569) at T0-hours (*p* = 0.05) (Table 3). The hyaluronan and microcirculatory profiles over 48 h are shown in Fig. 4.

**Fig. 5:**
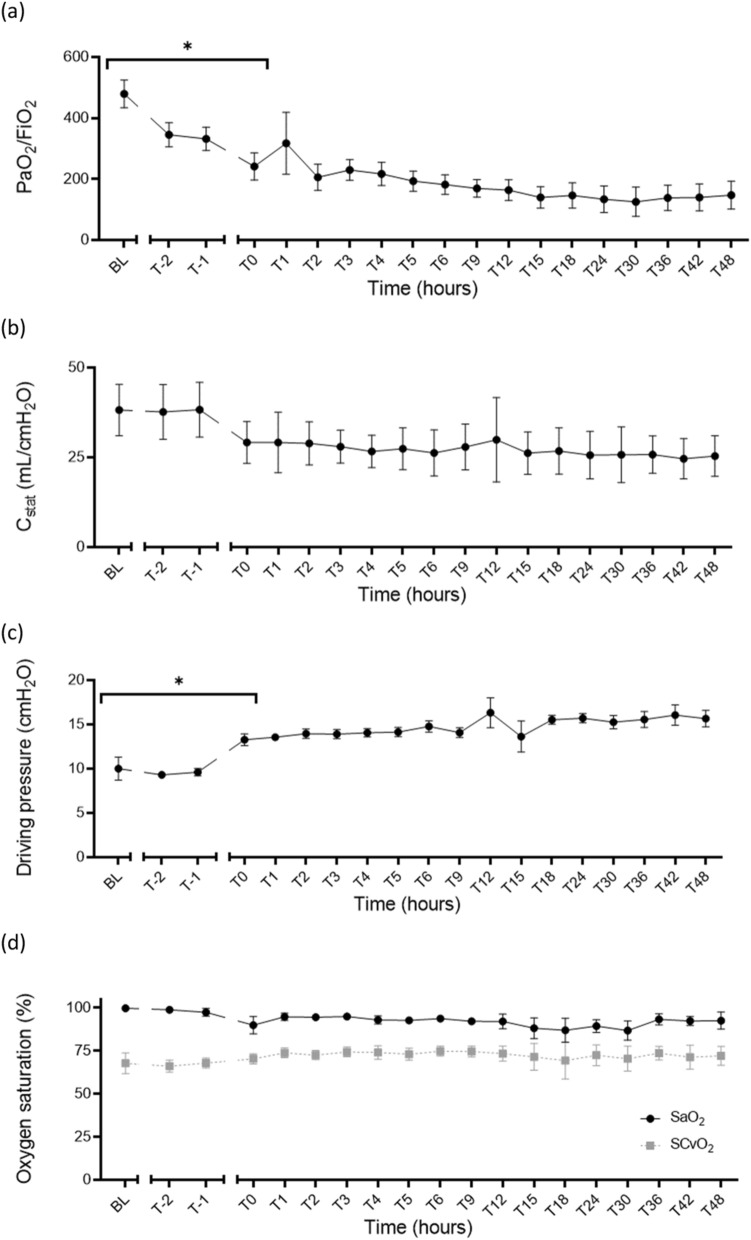
Development of septic shock (T0-hours) was characterised by significant changes in the mechanical ventilation with a reduction in the median PaO_2_/FiO_2_ from 492 (413, 538) at baseline to 186 (117, 189) (*p* = 0.03) (**a**); and the median static lung compliance from 38 mL/cmH_2_O (31, 45) at baseline to 29 mL/cmH_2_O (25, 31) (*p* = 0.036) (**b**). The median pulmonary driving pressure increased from 8.9 cmH_2_O (8.4, 12.73) at baseline to 12.7 cmH_2_O (12.0, 14.9) (*p* = 0.03) (**c**). Changes in the SaO_2_ and SvO_2_ were however not significant (*p* = 0.62 and *p* = 0.88, respectively) (**d**). *p < 0.05; *SaO*_*2*_ arterial oxygen saturation, *SvO*_*2*_ mixed venous oxygen saturation, *BL* baseline, *T-2* end of instrumentation, *T-1* start of bacterial infusion, *T0* diagnosis of shock
  (ii) Hemodynamic parameters
    Induction of septic shock led to a significant change from baseline in the median heart rate from 87/min, (76, 93) to 108/min (96, 114) (*p* = 0.02); the systemic vascular resistance index, SVRI from 1348 dynes s m^2^/cm^5^ (1268, 1448) to 676 dynes s m^2^/cm^5^ (483, 1025) (*p* < 0.0001); the mean pulmonary artery pressure from 9 mmHg (7, 10) to 14 mmHg (14, 21) (*p* = 0.02) with corresponding increases in the pulmonary wedge pressure from 2 mmHg (2, 6) to 8 mmHg (7, 11) (*p* = 0.0041) (Table 2). The 48-h trends in the haemodynamic variables are shown in Fig. 6.

**Fig. 6:**
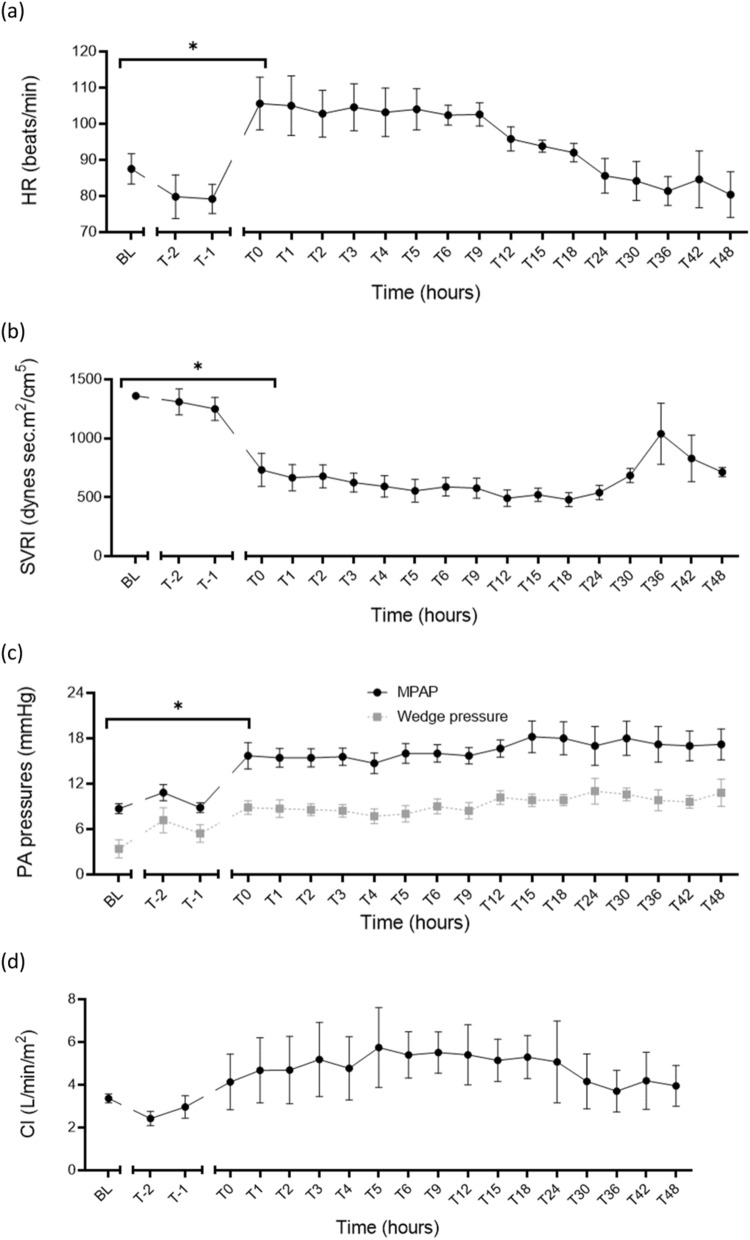
Physiological assessments showing septic shock induction (T0-hours) led to significant changes in the median heart rate from a median of 87/min (76, 93) to 108/min (96, 114) (*p* = 0.024) (**a**); SVRI from 1348 dynes s m^2^/cm^5^ (1268, 1448) to 676 dynes s m^2^/cm^5^ (483, 1025) (*p* < 0.0001) (**b**); MPAP from 9 mmHg (7, 10) to 14 mmHg (14, 21) (*p* = 0.02); and pulmonary wedge pressure from 2 mmHg (2, 6) to 8 mmHg (7, 10) (*p* = 0.0041) (**c**). The cardiac index from baseline to time of shock diagnosis (T0) did not change significantly (*p* = 0.99) (**d**). *p < 0.05; *HR* heart rate, *MPAP* mean pulmonary arterial blood pressure, *SVRI* systemic vascular resistance index, *BL* baseline, *T-2* end of instrumentation, *T-1* start of bacterial infusion, *T0* diagnosis of shock
- Laboratory assessments
  (i) Inflammatory cytokines
    The median baseline levels of circulating pro-inflammatory cytokines increased significantly following development of septic shock (T0-hours). Median IL-6 increased from 8 pg/mL (8, 49) to 12,494 pg/mL (3143, 18,867) (*p* < 0.01); IL-8 increased from 236 pg/mL (160, 432) to 425 pg/mL (368, 739) (*p* < 0.01); and VEGFA increased from 9 pg/mL (1, 34) to 101 pg/mL (30, 116) (*p* < 0.01). The anti-inflammatory cytokine IL-10 also increased from a baseline median of 36 pg/mL (22, 146) to 1433 pg/mL (513, 2212) (*p* < 0.01) (Table 3). The 48-h trends of the inflammatory cytokines are shown in Fig. 7. Inflammatory cytokine levels in the pulmonary bronchoalveolar lavage fluid showed features consistent with inflammation and are presented in Supplemental Table 4

**Fig. 7:**
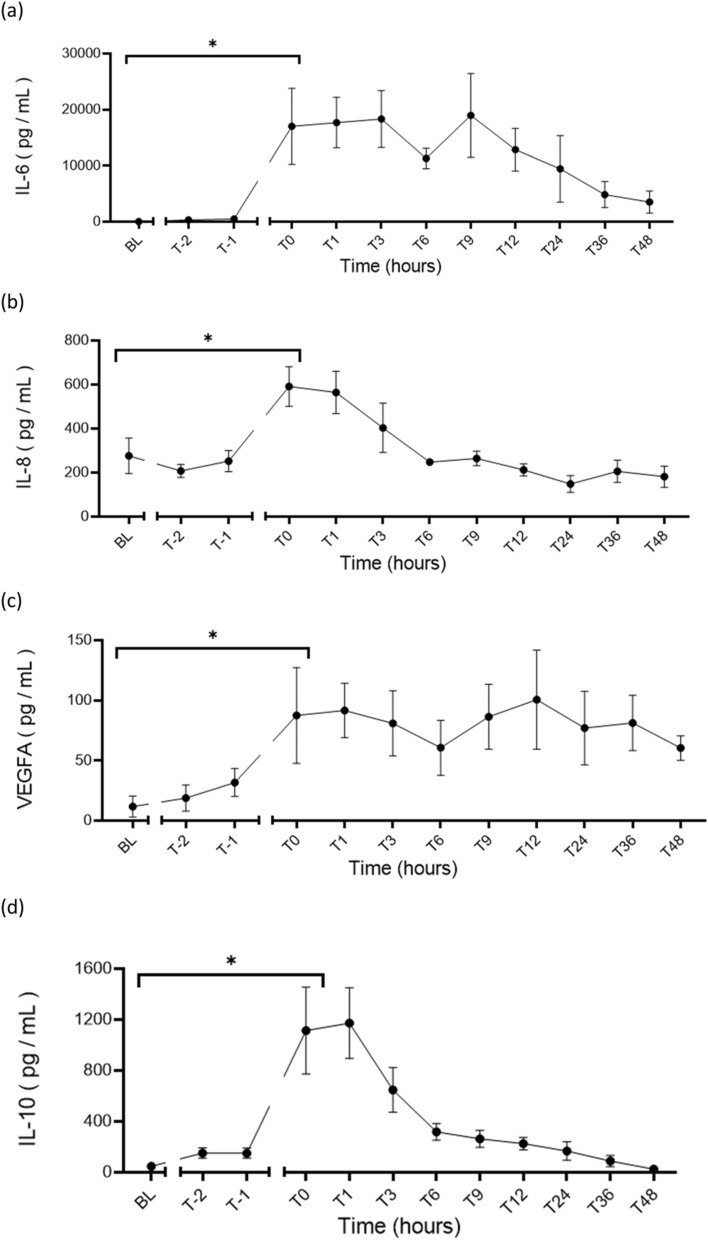
Inflammatory cytokines in serum showing significant increases from baseline following development of septic shock the pro-inflammatory cytokines: IL-6 (*p* < 0.01) (**a**) IL-8 (*p* < 0.01) (**b**); and VEGFA (*p* < 0.01) (**c**); as well as the anti-inflammatory cytokine IL-10 (< 0.01) (**d**). *p < 0.05; *IL* interleukin, *VEGFA* vascular endothelial growth factor A, *BL* baseline, *T-2* end of instrumentation, *T-1* start of bacterial infusion, *T0* diagnosis of shock
  (ii) Coagulopathy
    Induction of septic shock led to significant reduction in the levels of anti-thrombin, protein C and protein S (*p* < 0.01) with an increase in the activated partial thromboplastin time (aPTT) (*p* = 0.02). Rotational thromboelastometry testing showed non-significant prolongation of the clotting time on EXTEM (*p* = 0.07), INTEM (*p* = 0.36) and FIBTEM (*p* = 0.08). There was a reduction in platelet function on multiplate testing but this did not reach statistical threshold for either the adenine diphosphate (*p* = 0.42) or collagen (*p* = 0.41) tests (Table 3).
  (iii) Organ assessment biomarkers and microdialysis
    *Heart muscle:* Induction of septic shock led to a significant increase in the circulating levels of cardiac troponin I (*p* = 0.03), atrial-type natriuretic peptide (*p* = 0.02) and creatine kinase (*p* < 0.01) (Table 3). However, the increase in the baseline lactate in the heart from 1.7 mM (0.7, 3.0) to 3.1 mM (2.7, 3.3) after sepsis induction was non-significant (*p* = 0.16) (Fig. 8a).

**Fig. 8:**
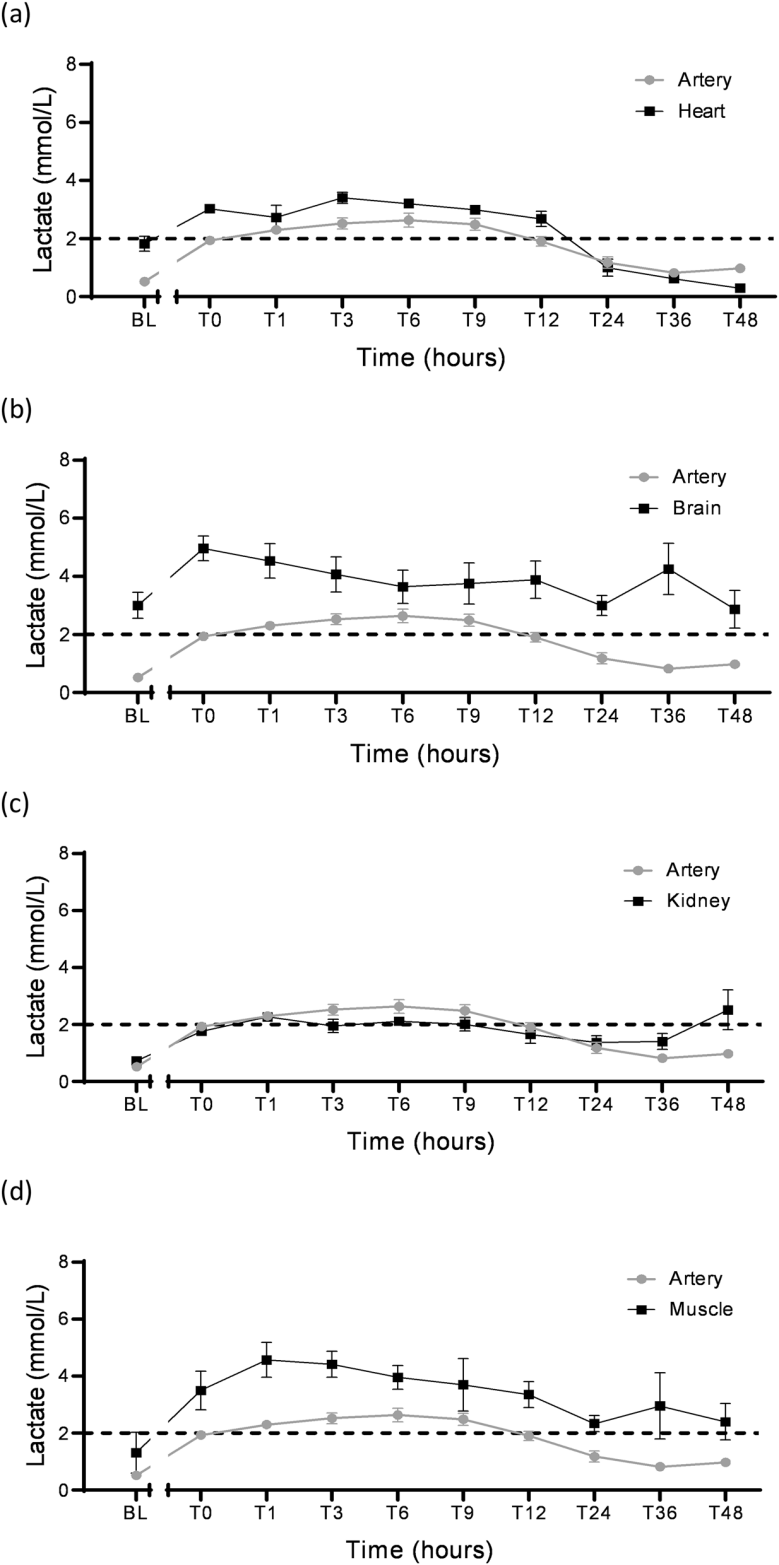
Microdialysis results showing tissue lactate levels with arterial lactate superimposed and the dotted line indicating the cut-off level of 2 mmol/L used for septic shock diagnosis. Induction of septic shock led to a non-significant increase in the median baseline lactate levels in the heart from 1.7 mM (0.7, 3.0) to 3.1 mM (2.6, 3.5) (*p* = 0.16) (**a**); in the brain from 3.5 mM (0.7, 5.1) to 5.6 mM (2.9, 6.7) (*p* = 0.48) (**b**); in the kidney from 0.8 mM (0.6, 0.8) to 1.8 mM (1.2, 2.3) (*p* = 0.13) (**c**); and in the muscle from 1.0 mM (0.03, 3.8) to 3.6 mM (1.4, 5.5) (*p* = 0.26) (**d)**. *BL* baseline, *T0* diagnosis of shock
    *Brain:* The median lactate levels in the brain at baseline were elevated 3.5 mM (0.8, 5.1) and increased to 5.6 mM (2.9, 6.7) after induction of septic shock (*p* = 0.48) (Fig. 8b).
    *Kidney:* After sepsis induction, neither the reduction in urine output (*p* = 0.99) (Table 2), nor the mild increase in creatinine levels (*p* = 0.99) (Table 3) was significant. The median lactate levels in the kidney were low at baseline 0.78 mM (0.57, 0.8) and increased to 1.8 mM (1.2, 2.3) after induction of septic shock, but this was not significant (*p* = 0.13) (Fig. 8c).
    *Liver:* There was a significant reduction in the total protein (*p* < 0.01), albumin (*p* < 0.01) and gamma-glutamyl transferase (*p* = 0.03) following induction of septic shock (Table 3). There were no microdialysis catheters inserted in the liver.
    *Skeletal muscle:* The median lactate levels in the skeletal muscle at baseline was 1.0 mM (0.03, 3.8) and increased to 3.6 mM (1.4, 5.5), but this was not significant (*p* = 0.26) after induction of septic shock (Fig. 8d).
- Tissue histopathology and transmission electron microscopy
  The histological assessment showed acute inflammatory features including neutrophilic infiltration, congestion, and oedema consistent with septic shock in the heart, lungs, kidney, brain, and liver. Similarly, transmission electron microscopy revealed early degenerative changes mainly in the endothelial cells and some parenchymal cells in these organs. However, parenchymal tissue assessment in the brain was not possible. A summary of the tissue histology scores, and transmission electron microscopy are presented in Table 4 and Supplemental Table 5, respectively.

**Table 4:** Tissue histology assessment and scoring

	Tissue score				
	Left upper lobe	Left lower lobe	Right upper lobe	Right middle lobe	Right lower lobe
(a) Lungs					
Intra-alveolar haemorrhage	0	0	0	0	0
Alveolar oedema	0.3	0.3	0.2	0.3	0.4
Neutrophils in the alveolar space	0.1	0.1	0.3	0	0.1
Neutrophils in the interalveolar capillaries and interstitial space	0.3	0.4	0.4	0.3	0.4
Thrombi within capillaries/small blood vessels	0	0	0	0	0
Hyaline membrane	0	0	0	0	0
Congestion	0.5	0.6	0.6	0.6	0.7

	Left ventricle	Right ventricle	Septum		
(b) Heart					
Contraction band necrosis	0.04	0.113	0.1		
Neutrophilic infiltration	0.013	0	0		
Haemorrhage	0.050	0.025	0		
Oedema	0	0	0		
Necrosis	0.013	0.090	0		
Vacuolation/myocytolysis	0.113	0.163	0.1		
Vasculitis	0	0	0		
Capillary fibrin thrombi	0	0	0		

	Cortex				
(c) Kidney					
Fibrin thrombi within the capillary loops	0				
Accumulation of granular material in Bowman’s space	0.4				
Tubular necrosis—proximal and distal tubules (cortex)	0				
Granular material or hyaline casts within the tubular lumen—proximal and distal tubules (cortex)	0.6				
Cloudy swelling/hydropic degeneration of tubular epithelial cells—proximal and distal tubules (cortex)	0.2				
Acute inflammation	0				
Congestion	0.1				
Haemorrhage	0.1				
(d) Brain					
Neuronal shrinkage and hyperacidophilia	0				
Spongy state	0.3				
Congestion	1.8				
Perivascular oedema	0.5				
Perivascular/interstitial haemorrhage	0.1				
Neutrophilic infiltration	0				
Capillary fibrin thrombi	0				
(e) Liver					
Sinusoidal congestion	0.1				
Haemorrhage	0				
Necrosis (random, centrilobular, or midzonal	0				
Single cell necrosis/apoptosis	0				
Neutrophils within sinusoids	0.2				
Hepatocyte vacuolation	0.4

## Discussion

We developed a reproducible septic shock model using a clinically relevant live bacterial infusion consistent with the recent Minimum Quality Threshold in Preclinical Sepsis Studies (MQTiPSS) recommendations [1]. As host responses to sepsis can be varied, we used animals of similar weight in the optimisation and model development experiments to standardise experimental conditions and minimise heterogeneity. Once bacterial dose and infusion-rate optimization was achieved, all sheep developed septic shock (as per Sepsis 3 criteria) within 12 h of completion of *E. coli* infusion, with corresponding increases in the vasopressor requirement (VDI) and cytokines (IL-6, IL-8, IL-10, and VEGFA). With advanced supportive intensive care therapy and active haemodynamic management, all the critically ill animals survived for 48 h after septic shock. Based on our data, this optimised model employing the *E. coli* ST131 strain EC958 use and an infectious dose of 2 × 10^7^ cfu/mL with a sequential hourly infusion-rate escalation caused reproducible septic shock. The volume restriction and early vasopressor resuscitation strategy applied over 48 h in this model preserved microcirculatory function without further increase in release of hyaluronan or worsening of the microcirculatory parameters over time. Tissue histopathological changes seen in endothelial cells were consistent with those described in the literature following sepsis [30]. However, electron microscopy revealed early degenerative changes in endothelial and parenchymal cells that are non-specific for sepsis but consistent with cellular stress or damage [31].

Although clinical sepsis definitions are regularly updated, they have not been historically aligned with the definitions employed in animal sepsis models. Hence, there has been limited clinical translation of hemodynamic resuscitation research conducted using animal models [25, 32, 33]. The MQTiPSS recommendations updated the definitions of animal sepsis models, recommended use of live bacterial (or fungal) strains from clinical isolates to induce sepsis and the subsequent application of treatments comparable to the clinical guidelines [1, 2].

We previously developed a hyperdynamic large animal (ovine) LPS-endotoxaemic shock model and demonstrated a paradoxical increase in vasopressor requirements in sheep receiving bolus fluid challenges [34, 35]. However, this LPS model was only physiologically supported by an escalating vasopressor dose without fluid resuscitation for up to 12-h post-endotoxemia. The downstream harmful effects of acute resuscitation in septic shock have been shown to occur after 24–48 h; hence, there is a need for models that replicate the complexity of clinical sepsis and survive beyond 12 h.

The most promising large animal bacterial sepsis models are ovine and porcine models due to their similarity to human physiology [36–38]. Trimmel et al. demonstrated that baseline carotid and femoral MAP measurements in mature sheep (2–5 years) were comparable to those in humans [39]. MAP is particularly important as it is one of the Sepsis-3 diagnostic criteria for septic shock and also forms a clinically relevant target for guiding resuscitation [25]. In some of the existing live pathogen infection models, a relatively large bacterial inoculum is administered to induce sepsis over a relatively short period of time, thus constituting a model of acute intoxication rather than evolving infection [40]. Other limitations of existing animal models of live pathogen infections include generalisability of findings obtained from a single organism strain, varying infection doses and administration rates among others. The lethality of live bacterial sepsis is dependent on the dose, route of administration, and concomitant antibiotic use. It is possible that the lethal dose administered may represent a state of fulminant endotoxemia with a cytokine storm rather than sepsis [40].

Some large animal models of live bacterial sepsis do not reproduce the severe critical illness seen clinically with patients undergoing anaesthesia and mechanical ventilation in the intensive care unit. Lankadeva et al. described a live bacterial ovine sepsis model induced by an *E. coli* strain isolated from the blood culture of a patient with sepsis. However, in this model, while the bacterial species used is clinically relevant, the animals underwent staged instrumentation with prolonged recovery periods (2–3 weeks) and the animals were conscious with a MAP consistently above 65 mmHg, thus limiting utility in intensive care resuscitation research [24, 41]. Increasingly, more clinically relevant models are required to investigate endothelial and microvascular responses to sepsis and resuscitation. Yini et al. described an *E. coli* septic shock canine model [42]. This model established a lethal bacterial dose by continuous infusion of *E. coli* through the external jugular vein for 2 h and showed that glycocalyx shedding was reduced following the therapeutic administration of unfractionated heparin. Skorup et al. developed an intensive care *E. coli* sepsis porcine model to investigate the effects of antibiotics on bacterial virulence and host response. Notably, similar to our study, the bacterial strain used in their study was isolated from a patient with bloodstream infection and had known sensitivity and resistance patterns. However, in contrast to our model, they used a randomized infusion of either killed or live *E. coli* bacteria (B09-11822 strain) to induce sepsis over a shorter period (3 h), with the MAP in the pigs investigated being high (78–100 mmHg) and thus not relevant for acute resuscitation research [43]. Jacquet-Lagrèze et al. also described a porcine septic shock model induced by infusion of *Pseudomonas aeruginosa*, but there was no significant reduction in the baseline MAP, making it unsuitable for acute resuscitation studies [38]. Furthermore, although *P. aeruginosa* is an important sepsis pathogen, its disease burden is approximately six times lower than *E. coli* [5].

### Strengths and limitations

We have developed a model for investigating the acute-phase pathophysiological effects of septic shock and resuscitation with strengths that include (within a single model):

- A large animal model of live bacterial distributive shock with active haemodynamic and intensive care management of the acute phase of illness. This is one of the few acutely ill large-animal models in which monitoring, and observations were extended to 48 h with a shorter post-instrumentation rest period.
- The duration of sepsis induction has been reproducible and acute within hours (6–12 h) unlike previously described chronic models [41] that also used LPS rather than live pathogens [44]. This makes the model ideal for evaluation of the acute-phase pathophysiology of endothelial dysfunction and end-organ failure resulting from septic shock and subsequent resuscitation administered. In addition, standardisation of the study protocol is an important step towards ensuring clinical translation.
- The clinical relevance of our model is further exemplified by the use of live pathogenic *E. coli* bacteria (ST131 strain EC958), which belongs to a clinically relevant multidrug-resistant *E. coli* lineage that causes high rates of human urinary tract and bloodstream infections [45–47]. *E. coli* strain EC958 has been completely sequenced [48] and is a prototype ST131 reference strain [20, 49–51]; thus, reproduction of acute critical illness using EC958 makes this model ideal for investigation of emergency and intensive care resuscitative as well as novel therapies. Our report could inform work from other groups and thus aid in employing the reduction, replacement, and refinement (3Rs) ethical strategies for conducting animal experiments.

As a limitation of most large animal models, our sample size was small, which could result in the heterogeneity of effects. However, the results concur with those of other comparable model-development experiments. We did not measure endotoxin levels to ascertain their release during bacterial infection, which could be tested in future modifications of the model.

## Conclusions

We have developed a reproducible large animal model of live bacterial septic shock. Our model represents a well-defined and appropriate tool for testing advanced therapeutic interventions. Together, the use of a prototype *E. coli* ST131 sepsis-causing reference strain and the characterized inflammatory cytokine profile make this model relevant for the future investigation of sepsis-induced host immune responses and the identification of host biomarkers to characterize disease progression.

## Supplementary Information

Supplementary material 1.

## Data Availability

All data generated or analysed during this study are included in this published article and its supplementary information files.

## Abbreviations

3Rs: Reduce, replace, and refine
ANOVA: Analysis of variance
CCRG: Critical Care Research Group
cfu: Colony forming units
CI: Cardiac index
CVP: Central venous pressure
*E. coli*: *Escherichia coli*
ELISA: Enzyme-linked immunosorbent assay
ETCO_2_: End-tidal carbon dioxide
FiO_2_: Fraction of inspired oxygen
HR: Heart rate
ICU: Intensive Care Unit
IL: Interleukin
IQR: Interquartile range
LPS: Lipopolysaccharide
MAP: Mean arterial blood pressure
MPAP: Mean pulmonary arterial pressure
MQTiPSS: Minimum Quality Threshold in Preclinical Sepsis Studies
*P. aeruginosa*: *Pseudomonas aeruginosa*
PaCO_2_: Arterial partial pressure of carbon dioxide
PaO_2_: Arterial partial pressure of oxygen
PAMPs: Pathogen-associated molecular patterns
*P*/*F * ratio: Ratio of arterial partial pressure of oxygen/fraction of inspired oxygen
PEEP: Positive end-expiratory pressure
PPV: Proportion of perfused vessels
PVD: Perfused vessel density
QUT–MERF: Queensland University of Technology–Medical Engineering Research Facility
SaO_2_: Arterial oxygen saturation
ScvO_2_: Central venous oxygen saturation
SD: Standard deviation
SSC: Surviving Sepsis Campaign
ST: Sequence type
SVRI: Systemic vascular resistance index
TVD: Total vessel density
UQ: University of Queensland
VDI: Vasopressor dependency index
VEGFA: Vascular endothelial growth factor A
VIS: Vasoactive inotrope score
WBC: White blood cell
WHO: World Health Organization

## References

[1] Libert C, Ayala A, Bauer M, Cavaillon JM, Deutschman C, Frostell C et al (2019) Part II: minimum quality threshold in preclinical sepsis studies (MQTiPSS) for types of infections and organ dysfunction endpoints. Shock 51(1):23–32 30106873 10.1097/SHK.0000000000001242 PMC6296883

[2] Osuchowski MF, Ayala A, Bahrami S, Bauer M, Boros M, Cavaillon JM et al (2018) Minimum quality threshold in pre-clinical sepsis studies (MQTiPSS): an international expert consensus initiative for improvement of animal modeling in sepsis. Shock 50(4):377–380 30106875 10.1097/SHK.0000000000001212 PMC6133201

[3] Mu A, Klare WP, Baines SL, Ignatius Pang CN, Guerillot R, Harbison-Price N et al (2023) Integrative omics identifies conserved and pathogen-specific responses of sepsis-causing bacteria. Nat Commun 14(1):1530 36934086 10.1038/s41467-023-37200-w PMC10024524

[4] Laupland KB (2013) Incidence of bloodstream infection: a review of population-based studies. Clin Microbiol Infect 19(6):492–500 23398633 10.1111/1469-0691.12144

[5] Coombs GBJ, Daley D, Collignon P, Cooley L, Gottlieb T, Iredell J, Kotsanas D, Nimmo G, Robson J, on behalf of the Australian Group on Antimicrobial Resistance and Australian Commission on Safety and Quality in Health Care (2019) Sepsis outcome programs. Australian Group on Antimicrobial Resistance

[6] Umemura Y, Ogura H, Takuma K, Fujishima S, Abe T, Kushimoto S et al (2021) Current spectrum of causative pathogens in sepsis: a prospective nationwide cohort study in Japan. Int J Infect Dis 103:343–351 33221519 10.1016/j.ijid.2020.11.168

[7] Remick DG, Ayala A, Chaudry IH, Coopersmith CM, Deutschman C, Hellman J et al (2019) Premise for standardized sepsis models. Shock 51(1):4–9 29877959 10.1097/SHK.0000000000001164 PMC6281773

[8] Marshall JC (2010) From the bedside back to the bench: the role of preclinical studies in understanding clinical therapies. Crit Care Med 38(1):329–330 20023488 10.1097/CCM.0b013e3181b9d4b4

[9] Rivers E, Nguyen B, Havstad S, Ressler J, Muzzin A, Knoblich B et al (2001) Early goal-directed therapy in the treatment of severe sepsis and septic shock. N Engl J Med 345(19):1368–1377 11794169 10.1056/NEJMoa010307

[10] Maitland K, Kiguli S, Opoka RO, Engoru C, Olupot-Olupot P, Akech SO et al (2011) Mortality after fluid bolus in African children with severe infection. N Engl J Med 364(26):2483–2495 21615299 10.1056/NEJMoa1101549

[11] Pro CI, Yealy DM, Kellum JA, Huang DT, Barnato AE, Weissfeld LA et al (2014) A randomized trial of protocol-based care for early septic shock. N Engl J Med 370(18):1683–1693 24635773 10.1056/NEJMoa1401602 PMC4101700

[12] Mouncey PR, Osborn TM, Power GS, Harrison DA, Sadique MZ, Grieve RD et al (2015) Trial of early, goal-directed resuscitation for septic shock. N Engl J Med 372(14):1301–1311 25776532 10.1056/NEJMoa1500896

[13] ARISE Investigators, ANZICS Clinical Trials Group, Peake SL, Delaney A, Bailey M, Bellomo R et al (2014) Goal-directed resuscitation for patients with early septic shock. N Engl J Med 371(16):1496–1506 25272316 10.1056/NEJMoa1404380

[14] Marik PE, Byrne L, van Haren F (2020) Fluid resuscitation in sepsis: the great 30 mL per kg hoax. J Thorac Dis 12(Suppl 1):S37–S47 32148924 10.21037/jtd.2019.12.84 PMC7024756

[15] Ospina-Tascon GA, Hernandez G, Alvarez I, Calderon-Tapia LE, Manzano-Nunez R, Sanchez-Ortiz AI et al (2020) Effects of very early start of norepinephrine in patients with septic shock: a propensity score-based analysis. Crit Care 24(1):52 32059682 10.1186/s13054-020-2756-3 PMC7023737

[16] Permpikul C, Tongyoo S, Viarasilpa T, Trainarongsakul T, Chakorn T, Udompanturak S (2019) Early use of norepinephrine in septic shock resuscitation (CENSER). A randomized trial. Am J Respir Crit Care Med 199(9):1097–1105 30704260 10.1164/rccm.201806-1034OC

[17] Meyhoff TS, Hjortrup PB, Wetterslev J, Sivapalan P, Laake JH, Cronhjort M et al (2022) Restriction of intravenous fluid in ICU patients with septic shock. N Engl J Med 386(26):2459–2470 35709019 10.1056/NEJMoa2202707

[18] (2013) Australian code for the care and use of animals for scientific purposes, 8th edn

[19] Chemonges S, Shekar K, Tung JP, Dunster KR, Diab S, Platts D et al (2014) Optimal management of the critically ill: anaesthesia, monitoring, data capture, and point-of-care technological practices in ovine models of critical care. Biomed Res Int 2014:468309 24783206 10.1155/2014/468309 PMC3982457

[20] Phan MD, Peters KM, Alvarez Fraga L, Wallis SC, Hancock SJ, Nhu NTK et al (2022) Plasmid-mediated ciprofloxacin resistance imparts a selective advantage on *Escherichia coli* ST131. Antimicrob Agents Chemother 66(1):e0214621 34780264 10.1128/AAC.02146-21 PMC8765324

[21] Phan MD, Peters KM, Sarkar S, Forde BM, Lo AW, Stanton-Cook M et al (2015) Third-generation cephalosporin resistance conferred by a chromosomally encoded blaCMY-23 gene in the *Escherichia coli* ST131 reference strain EC958. J Antimicrob Chemother 70(7):1969–1972 25786480 10.1093/jac/dkv066

[22] Schembri MA, Zakour NL, Phan MD, Forde BM, Stanton-Cook M, Beatson SA (2015) Molecular characterization of the multidrug resistant *Escherichia coli* ST131 clone. Pathogens 4(3):422–430 26131613 10.3390/pathogens4030422 PMC4584265

[23] See Hoe LE, Wildi K, Obonyo NG, Bartnikowski N, McDonald C, Sato K et al (2021) A clinically relevant sheep model of orthotopic heart transplantation 24 h after donor brainstem death. Intensive Care Med Exp 9(1):60 34950993 10.1186/s40635-021-00425-4 PMC8702587

[24] Lankadeva YR, Peiris RM, Okazaki N, Birchall IE, Trask-Marino A, Dornom A et al (2020) Reversal of the pathophysiological responses to gram-negative sepsis by megadose vitamin C. Crit Care Med 49(2):e179–e190 10.1097/CCM.0000000000004770 PMC7803449 33239507

[25] Singer M, Deutschman CS, Seymour CW, Shankar-Hari M, Annane D, Bauer M et al (2016) The third international consensus definitions for sepsis and septic shock (sepsis-3). JAMA 315(8):801–810 26903338 10.1001/jama.2016.0287 PMC4968574

[26] Mackenzie DC, Noble VE (2014) Assessing volume status and fluid responsiveness in the emergency department. Clin Exp Emerg Med 1(2):67–77 27752556 10.15441/ceem.14.040 PMC5052829

[27] Obonyo NG, Fanning JP, Ng AS, Pimenta LP, Shekar K, Platts DG et al (2016) Effects of volume resuscitation on the microcirculation in animal models of lipopolysaccharide sepsis: a systematic review. Intensive Care Med Exp 4(1):38 27873263 10.1186/s40635-016-0112-3 PMC5118377

[28] Amaro R, Li Bassi G, Motos A, Fernandez-Barat L, Aguilera Xiol E, Rigol M et al (2021) Development and characterization of a new swine model of invasive pneumococcal pneumonia. Lab Anim 50(11):327–335 10.1038/s41684-021-00876-y 34675433

[29] McIntosh AM, Tong S, Deakyne SJ, Davidson JA, Scott HF (2017) Validation of the vasoactive-inotropic score in pediatric sepsis. Pediatr Crit Care Med 18(8):750–757 28486385 10.1097/PCC.0000000000001191 PMC5548505

[30] Stassi C, Mondello C, Baldino G, Ventura Spagnolo E (2020) Post-mortem investigations for the diagnosis of sepsis: a review of literature. Diagnostics 10(10):849 33092081 10.3390/diagnostics10100849 PMC7590167

[31] Garofalo AM, Lorente-Ros M, Goncalvez G, Carriedo D, Ballen-Barragan A, Villar-Fernandez A et al (2019) Histopathological changes of organ dysfunction in sepsis. Intensive Care Med Exp 7(Suppl 1):45 31346833 10.1186/s40635-019-0236-3 PMC6658642

[32] Weiss SL, Peters MJ, Alhazzani W, Agus MSD, Flori HR, Inwald DP et al (2020) Surviving sepsis campaign international guidelines for the management of septic shock and sepsis-associated organ dysfunction in children. Pediatr Crit Care Med 21(2):e52–e106 32032273 10.1097/PCC.0000000000002198

[33] Evans L, Rhodes A, Alhazzani W, Antonelli M, Coopersmith CM, French C et al (2021) Surviving sepsis campaign: international guidelines for management of sepsis and septic shock 2021. Crit Care Med 49(11):e1063–e1143 34605781 10.1097/CCM.0000000000005337

[34] Byrne L, Obonyo NG, Diab SD, Dunster KR, Passmore MR, Boon A-C et al (2018) Unintended consequences; fluid resuscitation worsens shock in an ovine model of endotoxemia. Am J Respir Crit Care Med. 10.1164/rccm.201801-0064OC 29882682 10.1164/rccm.201801-0064OC PMC7613331

[35] Byrne L, Obonyo NG, Diab S, Dunster K, Passmore M, Boon AC et al (2018) An ovine model of hyperdynamic endotoxemia and vital organ metabolism. Shock 49(1):99–107 28520696 10.1097/SHK.0000000000000904 PMC7004818

[36] DiVincenti L Jr, Westcott R, Lee C (2014) Sheep (*Ovis aries*) as a model for cardiovascular surgery and management before, during, and after cardiopulmonary bypass. J Am Assoc Lab Anim Sci 53(5):439–448 25255065 PMC4181684

[37] Barnhart GR, Jones M, Ishihara T, Chavez AM, Rose DM, Ferrans VJ (1982) Bioprosthetic valvular failure. Clinical and pathological observations in an experimental animal model. J Thorac Cardiovasc Surg 83(4):618–631 7062773

[38] Jacquet-Lagreze M, Allaouchiche B, Restagno D, Paquet C, Ayoub JY, Etienne J et al (2015) Gut and sublingual microvascular effect of esmolol during septic shock in a porcine model. Crit Care 19(1):241 26041462 10.1186/s13054-015-0960-3 PMC4490718

[39] Trimmel NE, Podgorsak A, Oertel MF, Jucker S, Arras M, Schmid Daners M et al (2022) The sheep as a comprehensive animal model to investigate interdependent physiological pressure propagation and multiparameter influence on cerebrospinal fluid dynamics. Front Neurosci 16:868567 35431780 10.3389/fnins.2022.868567 PMC9008349

[40] Poli-de-Figueiredo LF, Garrido AG, Nakagawa N, Sannomiya P (2008) Experimental models of sepsis and their clinical relevance. Shock 30(Suppl 1):53–59 18704008 10.1097/SHK.0b013e318181a343

[41] Lankadeva YR, Kosaka J, Evans RG, May CN (2018) An ovine model for studying the pathophysiology of septic acute kidney injury. Methods Mol Biol 1717:207–218 29468594 10.1007/978-1-4939-7526-6_16

[42] Yini S, Heng Z, Xin A, Xiaochun M (2015) Effect of unfractionated heparin on endothelial glycocalyx in a septic shock model. Acta Anaesthesiol Scand 59(2):160–169 25312742 10.1111/aas.12418

[43] Skorup P, Maudsdotter L, Lipcsey M, Larsson A, Sjolin J (2020) Mode of bacterial killing affects the inflammatory response and associated organ dysfunctions in a porcine *E. coli* intensive care sepsis model. Crit Care 24(1):646 33189146 10.1186/s13054-020-03303-9 PMC7666448

[44] Talke P, Dunn A, Lawlis L, Sziebert L, White A, Herndon D et al (1985) A model of ovine endotoxemia characterized by an increased cardiac output. Circ Shock 17(2):103–108 3902275

[45] Totsika M, Beatson SA, Sarkar S, Phan MD, Petty NK, Bachmann N et al (2011) Insights into a multidrug resistant *Escherichia coli* pathogen of the globally disseminated ST131 lineage: genome analysis and virulence mechanisms. PLoS ONE 6(10):e26578 22053197 10.1371/journal.pone.0026578 PMC3203889

[46] Petty NK, Ben Zakour NL, Stanton-Cook M, Skippington E, Totsika M, Forde BM et al (2014) Global dissemination of a multidrug resistant *Escherichia coli* clone. Proc Natl Acad Sci USA 111(15):5694–5699 24706808 10.1073/pnas.1322678111 PMC3992628

[47] Harris PNA, Ben Zakour NL, Roberts LW, Wailan AM, Zowawi HM, Tambyah PA et al (2018) Whole genome analysis of cephalosporin-resistant *Escherichia coli* from bloodstream infections in Australia, New Zealand and Singapore: high prevalence of CMY-2 producers and ST131 carrying blaCTX-M-15 and blaCTX-M-27. J Antimicrob Chemother 73(3):634–642 29253152 10.1093/jac/dkx466

[48] Forde BM, Ben Zakour NL, Stanton-Cook M, Phan MD, Totsika M, Peters KM et al (2014) The complete genome sequence of *Escherichia coli* EC958: a high quality reference sequence for the globally disseminated multidrug resistant *E. coli* O25b:H4-ST131 clone. PLoS ONE 9(8):e104400 25126841 10.1371/journal.pone.0104400 PMC4134206

[49] Ben Zakour NL, Alsheikh-Hussain AS, Ashcroft MM, Khanh Nhu NT, Roberts LW, Stanton-Cook M et al (2016) Sequential acquisition of virulence and fluoroquinolone resistance has shaped the evolution of *Escherichia coli* ST131. MBio 7(2):e00347-16 27118589 10.1128/mBio.00347-16 PMC4850260

[50] Totsika M, Kostakioti M, Hannan TJ, Upton M, Beatson SA, Janetka JW et al (2013) A FimH inhibitor prevents acute bladder infection and treats chronic cystitis caused by multidrug-resistant uropathogenic *Escherichia coli* ST131. J Infect Dis 208(6):921–928 23737602 10.1093/infdis/jit245 PMC3749003

[51] Phan MD, Peters KM, Sarkar S, Lukowski SW, Allsopp LP, Gomes Moriel D et al (2013) The serum resistome of a globally disseminated multidrug resistant uropathogenic *Escherichia coli* clone. PLoS Genet 9(10):e1003834 24098145 10.1371/journal.pgen.1003834 PMC3789825

