# Supplementary material for: An ovine septic shock model of live bacterial infusion
Source: Intensive Care Med Exp. 2024 Oct 28;12:94. doi: 10.1186/s40635-024-00684-x (PMC11519284; doi:10.1186/s40635-024-00684-x)
Supplement: Supplementary file 1 — Supplementary material 1. [file 40635_2024_684_MOESM1_ESM.docx]

**Supplemental Material**

**(1) Supplemental Methods**

At the end of the experiment of the 48-hour monitoring period, animals were euthanised (sodium pentobarbitone 0.5mg/kg) and tissue samples obtained for histopathology and transmission electron microscopy (table below):

| **Organ** | **Samples** | **Sections** |
| --- | --- | --- |
| Heart | Left and right ventricle, and septum | Longitudinal and transverse |
| Lung | - *Right lung*: Upper, lower and middle lobes - *Left lung*: Upper and lower lobes | Longitudinal and transverse |
| Kidney | Cortex | Longitudinal and transverse |
| Liver | Left lobe | Centri-lobar area |
| Brain | Left hemisphere | Frontal and parietal lobes |

*Histology*:

All samples were cut into slices ~5mm thickness after collection and fixed in 10% buffered formalin for 24-hours except for the brain samples which were fixed in formalin for 48-hours. Thereafter samples were put in embedding cassettes, rinsed and 70% ethanol added before being embedded in paraffin. Sections (4 µm) were stained with haematoxylin & eosin. Stained slides underwent bright field scanning at 40X magnification (up to 15 minutes scanning per slide), and thereafter histology scoring by an expert veterinary pathologist (blinded to the slide identity) (scoring table below):

| **Organ and scoring parameters** | **Scoring matrix** | | |
| --- | --- | --- | --- |
| **(a) Heart** | **0** | **1** | **2** |
| Contraction band necrosis | None | single fibre affected | multiple fibres |
| Neutrophilic infiltration | none | <20 neutrophils | >20 neutrophils |
| Haemorrhage | none | <50% affected | >50% affected |
| Oedema | none | <50% affected | >50% affected |
| Necrosis | Not present or single cell events | <50% affected | >50% affected |
| Vacuolation/myocytolysis | Not present or single cell events | <50% affected | >50% affected |
| Vasculitis | none | 1 vessel affected | >vessel affected |
| Capillary fibrin thrombi | none | 1 thrombus | >1 thrombus |
| **(b) Lungs** | **0** | **1** | **2** |
| Intra-alveolar haemorrhage | none | <50% of the alveoli | >50% of the alveoli |
| Alveolar oedema | none | <50% of the field | >50% of the field |
| Neutrophils in the alveolar space | none | 1-20 neutrophils | >20 neutrophils |
| Neutrophils in the interalveolar capillaries and interstitial space | none | 1-20 neutrophils | >20 neutrophils |
| Thrombi within capillaries/small blood vessels | none | 1 thrombus | >1 |
| Hyaline membrane | none | in 1 alveolus | in > 1 alveolus |
| Congestion | <2x | 2x-4x | >4x |
| **(c) Kidney** | **0** | **1** | **2** |
| Fibrin thrombi within the capillary loops | none | 1 thrombus | >1 |
| Accumulation of granular material in Bowman’s space | none | <50% glomeruli | >50% glomeruli |
| Tubular Necrosis – proximal and distal tubules (cortex) | none | <50% epithelial cells | > 50% epithelial cells |
| Granular material or hyaline casts within the tubular lumen – proximal and distal tubules (cortex) | none | <50% tubules | >50% tubules |
| Cloudy swelling/hydropic degeneration of tubular epithelial cells – proximal and distal tubules (cortex) | none | <50% epithelial cells | > 50% epithelial cells |
| Acute inflammation | none | <20 inflammatory cells | > 20 cells |
| Congestion | none | <50% tissue affected | > 50% tissue affected |
| Haemorrhage | none | <50% tissue affected | > 50% tissue affected |
| **(d) Brain** | **1** | **2** | **3** |
| Neuronal shrinkage and hyperacidophilia | none | <10 cells affected | >10 cells |
| Spongy state | none | <50% tissue | >50% tissue |
| Congestion | none | <50% of the field | >50% of the field |
| Perivascular oedema | none | <50% blood vessels | >50% blood vessels |
| Perivascular/interstitial haemorrhage | none | <50% blood vessels | >50% blood vessels |
| Neutrophilic infiltration | none | <20 neutrophils | >20 neutrophils |
| Capillary fibrin thrombi | none | 1 thrombus | >1 thrombus |
| **(e) Liver** | **0** | **1** | **2** |
| Sinusoidal congestion | None | <50% field | >50% field |
| Haemorrhage | None | <50% affected | >50% affected |
| Necrosis (random, centrilobular, or midzonal | Not present or single cell events | <50% affected | >50% affected |
| Single cell necrosis/apoptosis | None | <10 hepatocytes | >10 hepatocytes |
| Neutrophils within sinusoids | None | 1-20 neutrophils | >20 neutrophils |
| Hepatocyte vacuolation | None | <50% hepatocytes | >50% hepatocytes |

*Transmission electron microscopy (TEM)*

0.2M sodium cacodylate trihydrate pH 7.4 was diluted to 0.1M and mixed with 25% glutaraldehyde to prepare fixative solution. Wash buffer was prepared by mixing the fixative solution with a 2% stock solution of ruthenium red prepared in milliQ water. Tissue samples were cut into 10mm^3^ X 5mm^3^ sizes and immediately placed in a 1:1 mixture of the wash buffer and 4% paraformaldehyde (PFA) and stored at 4^0^C for up to 4 days after which the fixative solution was replaced with the wash buffer solution (5 minutes X 3 washes) and stored. Samples were transported to the imaging facility in 1X PBS, where they were further cut to 1mm^3^ X 1mm^3^, fixed with osmium tetroxide, dehydrated through graded ethanol, embedded in EPON812 resin and sectioned for TEM imaging (JEOL 2100 200 kV). Qualitative reporting of endothelial cells visualised on TEM was performed by an expert veterinary pathologist (assessment parameters in the table below):

|  | Electron microscopic features of sepsis | Present (√) / Absent (X) /Not assessable (N/A) |
| --- | --- | --- |
| (a) Heart | **(1) Endothelial cells** |  |
|  | Endothelial cell swelling/oedema/reduced electrodensity |  |
|  | Multi-focal loss of plasma membrane |  |
|  | Electrolucent cytoplasm/intracytoplasmic oedema |  |
|  | Mitochondrial swelling and loss of cristae |  |
|  | Rough endoplasmic reticulum dilation/swelling |  |
|  | Membranous bodies present (remnants of organelles) |  |
|  | **(2) Interstitial space** |  |
|  | Interstitial oedema |  |
|  | Sarcoplasmic oedema |  |
|  | **(3) Myocardial cells** |  |
|  | Myofibril and intercalated disc structures |  |
|  | Intermyofibrillary oedema |  |
| (b) Lungs | **(1) Endothelial cells** |  |
|  | Endothelial cell swelling/oedema/reduced electrodensity |  |
|  | Multi-focal loss of plasma membrane |  |
|  | Electrolucent cytoplasm/intracytoplasmic oedema |  |
|  | Mitochondrial swelling and loss of cristae |  |
|  | Rough endoplasmic reticulum dilation/swelling |  |
|  | Membranous bodies present (remnants of organelles) |  |
|  | **(2) Epithelial cells** |  |
|  | Alveolar oedema |  |
| (c) Kidney | **(1) Endothelial cells** |  |
|  | Endothelial cell swelling/oedema/reduced electrodensity |  |
|  | Multi-focal loss of plasma membrane |  |
|  | Electrolucent cytoplasm/intracytoplasmic oedema |  |
|  | Mitochondrial swelling and loss of cristae |  |
|  | Rough endoplasmic reticulum dilation/swelling |  |
|  | Membranous bodies present (remnants of organelles) |  |
|  | **(2) Interstitial space** |  |
|  | Interstitial oedema |  |
|  | **(3) Tubular epithelial cells** |  |
|  | Mitochondrial swelling and clearing |  |
| (d) Brain | **(1) Endothelial cells** |  |
|  | Endothelial cell swelling/oedema/reduced electrodensity |  |
|  | Multi-focal loss of plasma membrane |  |
|  | Electrolucent cytoplasm/intracytoplasmic oedema |  |
|  | Mitochondrial swelling and loss of cristae |  |
|  | Rough endoplasmic reticulum dilation/swelling |  |
|  | Membranous bodies present (remnants of organelles) |  |
|  | **(2) Interstitial space** |  |
|  | Interstitial oedema |  |
|  | **(3) Neuronal cells** |  |
|  |  |  |

**(2) Supplemental Figures**

**Supplemental Figure S1: Fluid input and output status**

(a)

**Figure S1:** Fluid input and output status over time. All animals received up to 500mL to offset losses from overnight fasting and insensible loss during surgical instrumentation. After the onset of shock, the reduction in median urine output was not significant from a baseline value of 0.9mL/kg/hr (0.3, 1.8) to 0.44mL/kg/hr (0.3, 1.8) (p=0.99). The fluid input was increased after shock with a peak at T1 hours (p=0.72). The overall change in fluid input (F=2.06, p=0.32) and urine output (F=2.31, p=0.32) were not significant.

*BL, baseline; T-2, end of instrumentation; T-1, start of bacterial infusion; T0, diagnosis of shock.*

**Supplemental Figure S2: Potassium and sodium electrolyte levels**

(a)

(b)

**Figure S1:** Electrolyte levels showing electrolyte results for **(a)** potassium levels (the dotted line indicating the cut-off level of 5.5 mmol/L for severe hyperkalaemia). The median potassium levels increased from a baseline level of 3.7 mM (3.3, 3.8) to 4.9 mM (4.4, 5.3) at the time of shock confirmation (T0) (P=0.06). The peak potassium levels were seen at T6 (i.e., 4.9 mM (4.6, 5.4), but by T48, this had normalised (i.e., 4.4 mM (4.0, 4.7) with no significant differences in trend to T48 seen (p=0.97); and **(b)** sodium levels (the dotted line indicating the cut-off level of 145 mmol/L for hypernatraemia). There were no significant changes in the baseline sodium levels after induction of shock (p=0.82) or by the end of the monitoring period, T48 (p=0.66).

*BL, baseline; T-2, end of instrumentation; T0, diagnosis of shock.*

**Supplemental Figure S3: Echocardiography**

(a)

(b)

**Figure S3:** Echocardiography monitoring showing; **(a)** the change in ejection fraction was not a significant reduction from baseline at the time of shock confirmation (p=0.97) and the increase after resuscitation was also not significant (p=0.98); and **(b)** the fractional area change also did not change significantly after shock confirmation (p=0.97) or post resuscitation (p=0.99).

*BL, baseline; T0, diagnosis of shock; EF, ejection fraction; FAC, fractional area change.*

**Supplemental Figure S4: Haemoglobin and haematocrit levels**

(a)

(b)

**Figure S4:** **(a)** Graph showing the changes in haemoglobin levels from baseline after shock confirmation (T0) (P=0.47) and post-resuscitation (p=0.77) were not significant; and **(b)** Graph showing corresponding haematocrit levels with non-significant changes.

*BL, baseline; T-2, end of instrumentation; T-1, start of bacterial infusion; T0, diagnosis of shock.*

**(3) Supplemental Tables**

**Supplemental Table 1: Ventilation strategy**

| **Parameter** | **Value** |
| --- | --- |
| Mode | Volume controlled |
| Tidal Volume (V_t_) | Start at 8mL/kg, then reduce to 6 mL/kg |
| Respiratory Rate | ≤ 35 breaths per minute^a^ |
| FiO_2_ | Lowest required for SpO_2_ 88-95%^b^ |
| PEEP | Adjusted to maintain P_plat_ 28-30 cmH_2_O^c^, ideally increase PEEP from 5 cmH_2_0 to 8-10cmH_2_0 based on PCO_2_ |
| Plateau Pressure (P_plat_) | ≤ 30 cmH_2_O |
| I:E Ratio | 1:2 |
| **Supplemental Table S1:** Ventilation strategy  ^a^ Adjusted to maintain pH 7.30-7.45. Permissive hypercapnia may be tolerated to a minimum pH of ≥ 7.15.  ^b^ PaO_2_ 55-80 mmHg. If despite an FiO_2_ of 1.0 oxygenation targets are not met, PEEP may be increased to maintain P_plat_ ≤ 32 cmH_2_O.  ^c^ Total PEEP (extrinsic PEEP + intrinsic PEEP) should not exceed 20 cmH_2_O. PEEP may be reduced to 5 cmH_2_O to maintain P_plat_ ≤ 30 cm H_2_O. If despite a PEEP of 5 cmH_2_O, P_plat_ > 30 cmH_2_O, V_t_ may be reduced in 1 mL/kg steps until set at 4 mL/kg. | |

**Supplemental Table 2: Resuscitation Protocol**

| **Steps** | **Actions** |
| --- | --- |
| **1^st^** | If MAP falls <60mmHg for >5min  Increase maintenance from 1 mL/kg/hr to 2 mL/kg/hr and at the same time  start noradrenaline 0.05mcg/kg/min |
| **2^nd^** | Increase noradrenaline 0.02mcg/kg/min (every 5 min), if MAP <60mmHg   - maximum noradrenaline dose 0.2mcg/kg/min |
| **3^rd^** | Start vasopressin 0.6 units/hour (1.5 mL/hr), if MAP <60mmHg  Increase vasopressin by 0.2 units/hour (i.e., 0.5 mL/hr every 5 min)   - maximum vasopressin dose 2.4 units/hour (6ml~~s~~/hour) |
| **4^th^** | Fluid challenge (compound sodium lactate, CSL), if MAP <60mmHg   - 5 mL/kg in 15 min - Repeat x 6 (total volume 30mL/kg) |
| **Reassess:** | If MAP <60mmHg and any of:   1. Cardiac output <2L/m^2^ 2. Urine output <0.5ml/kg/hr 3. ABG lactate >2mmol/L |
| **5^th^** | Start adrenaline 0.05mcg/kg/min  Increase adrenaline 0.02mcg/kg/min (every 5 min), if MAP <60mmHg   - maximum adrenaline dose 0.2mcg/kg/min |
| **6^th^** | If MAP <60mmHg, give volume (CSL) 5mL/kg bolus X3   - target to maintain urine output ≥0.5mL/kg/hr) |
| **7^th^** | Reposition (i.e., Trendelenburg position)  Ensure all strategies to limit hypoxia have been addressed as per ventilatory strategy Supplemental Table 4. |
| **Hypoglycaemia Management** | Target blood sugar: >4 mmol/L   - Slow infusion 25% dextrose (1ml/hr) |
| **Supplemental Table S2:** Resuscitation protocol  *MAP, mean arterial blood pressure; ABG, arterial blood gas; CSL, compound sodium lactate (Hartmann’s) solution* | |

**Supplemental Table 3: De-Resuscitation Plan**

| **Steps** | **Actions** |
| --- | --- |
|  | Criteria for de-escalation:   1. MAP >60mmHg 2. Cardiac index >3L/min/m^2^ 3. Urine output >0.5mL/kg/hr |
| **1^st^** | Decrease maintenance from 2 mL/kg/hr to 1 mL/kg/hr |
| **2^nd^** | If on adrenaline, wean in 0.05mcg/kg/min aliquots - drop every hour if de-escalation criteria are achieved consistently |
| **3^rd^** | If on vasopressin, wean in 0.5mL/hour aliquots - drop every hour if de-escalation criteria are achieved consistently |
| **4^th^** | If on noradrenaline, wean in 0.05mcg/kg/min aliquots - drop every hour if de-escalation criteria are achieved consistently |
| **Supplemental Table S3:** De-Resuscitation protocol  *MAP, mean arterial blood pressure* | |

**Supplemental Table 4: Pulmonary bronchoalveolar lavage fluid assessment**

|  | **BL** | **T0** | **T48** | **p-value**  **(BL vs T0)** | **p-value**  **(T0 to Tend)** |
| --- | --- | --- | --- | --- | --- |
| **Cytokines** |  |  |  |  |  |
| IL-1β (pg/mL) | 47 (46, 47) | 70 (47, 185) | 572 (241, 989) | 0.99 | 0.03 |
| IL-6 (pg/mL)* | - | 312 (312, 313) | 590 (312, 1391) | - | 0.89 |
| IL-8 (pg/mL) | 163 (20, 396) | 598 (334, 884) | 1229 (1025, 1844) | 0.62 | 0.14 |
| IL-10 (pg/mL) | 125 (113, 125) | 125 (125, 226) | 133 (125, 961) | 0.99 | 0.46 |
| **Supplemental Table S4:** Pulmonary bronchoalveolar lavage fluid assessment.  Data presented as medians (IQR); BL, baseline; T0, diagnosis of shock; T48, time of experiment termination in hours; p-value (BL vs T0), significance test for effect of sepsis induction; p-value (T0 vs T48), significance test for effect of resuscitation.  *IL, interleukin.*  **Insufficient sample at baseline for analysis* | | | | | |

**Supplemental Table 5: Tissue transmission electron microscopy assessment**

|  | Electron microscopic features of sepsis | Present (√) / Absent (X) /Not assessable (N/A) |
| --- | --- | --- |
| (a) Heart | **(1) Endothelial cells** |  |
|  | Endothelial cell swelling/oedema/reduced electrodensity | √ |
|  | Multi-focal loss of plasma membrane | X |
|  | Electrolucent cytoplasm/intracytoplasmic oedema | √ |
|  | Mitochondrial swelling and loss of cristae | √ |
|  | Rough endoplasmic reticulum dilation/swelling | √ |
|  | Membranous bodies present (remnants of organelles) | X |
|  | **(2) Interstitial space** |  |
|  | Interstitial oedema | √ |
|  | Sarcoplasmic oedema | √ |
|  | **(3) Myocardial cells** |  |
|  | Myofibril and intercalated disc structures | √ |
|  | Intermyofibrillary oedema | √ |
| (b) Lungs | **(1) Endothelial cells** |  |
|  | Endothelial cell swelling/oedema/reduced electrodensity | √ |
|  | Multi-focal loss of plasma membrane | √ |
|  | Electrolucent cytoplasm/intracytoplasmic oedema | √ |
|  | Mitochondrial swelling and loss of cristae | √ |
|  | Rough endoplasmic reticulum dilation/swelling | √ |
|  | Membranous bodies present (remnants of organelles) | √ |
|  | **(2) Epithelial cells** |  |
|  | Alveolar oedema | √ |
| (c) Kidney | **(1) Endothelial cells** |  |
|  | Endothelial cell swelling/oedema/reduced electrodensity | √ |
|  | Multi-focal loss of plasma membrane | X |
|  | Electrolucent cytoplasm/intracytoplasmic oedema | X |
|  | Mitochondrial swelling and loss of cristae | √ |
|  | Rough endoplasmic reticulum dilation/swelling | √ |
|  | Membranous bodies present (remnants of organelles) | X |
|  | **(2) Interstitial space** |  |
|  | Interstitial oedema | N/A |
|  | **(3) Tubular epithelial cells** |  |
|  | Mitochondrial swelling and clearing | √ |
| (d) Brain | **(1) Endothelial cells** |  |
|  | Endothelial cell swelling/oedema/reduced electrodensity | √ |
|  | Multi-focal loss of plasma membrane | N/A |
|  | Electrolucent cytoplasm/intracytoplasmic oedema | √ |
|  | Mitochondrial swelling and loss of cristae | √ |
|  | Rough endoplasmic reticulum dilation/swelling | N/A |
|  | Membranous bodies present (remnants of organelles) | N/A |
|  | **(2) Interstitial space** |  |
|  | Interstitial oedema | N/A |
|  | **(3) Neuronal cells** | N/A |
| Supplemental Table S5: Transmission electron microscopic features of sepsis in tissues | | |
